# Supplementary material for: A mechanism for exocyst-mediated tethering via Arf6 and PIP5K1C-driven phosphoinositide conversion
Source: Curr Biol. Author manuscript; Available in PMC 2022 Sep 7. (PMC9382030; doi:10.1016/j.cub.2022.04.089)
Supplement: Supplementary Material [file EMS152831-supplement-Supplementary_Material.pdf]

Current Biology, Volume *32*

Supplemental Information

A mechanism for exocyst-mediated tethering via Arf6 and PIP5K1C-driven phosphoinositide conversion

Hannes Maib and David H. Murray

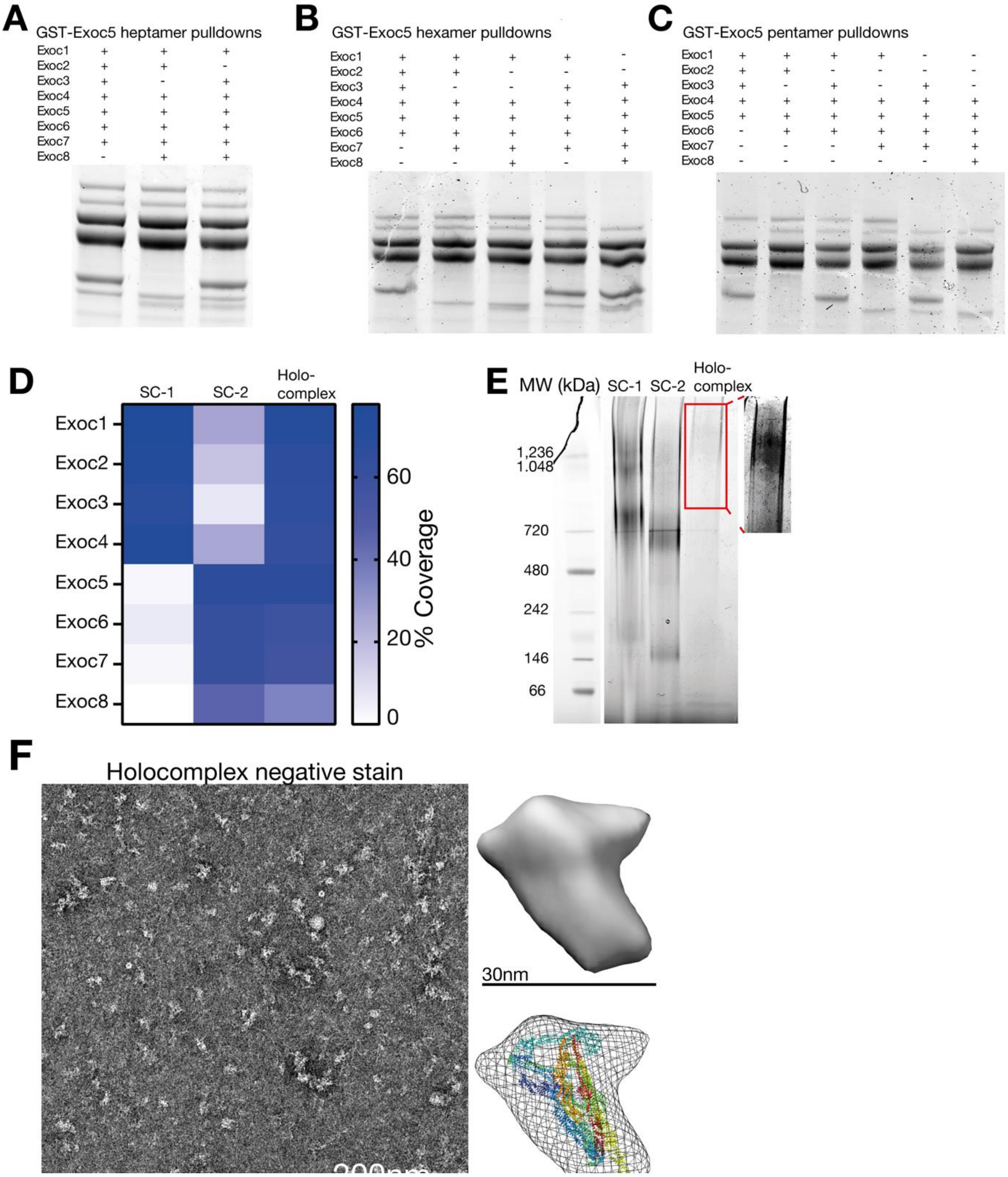

Figure S1. Exocyst connectivity and holocomplex verification and validation. Related to Figure 1
A-C, To confirm higher order exocyst complex connectivity, exocyst subunits were separately expressed in SF9 insect cells and mixed upon lysis. All possible combinations for exocyst hepta-, hexa-, and pentamers from pulldowns using Exoc5 tagged with GST and analysed by SDS-PAGE and Coomassie staining. **D**, Heatmap of protein coverage for mass spectrometry analysis of purified Subcomplex-1, -2 and holocomplex. **E**, Native gel electrophoresis of purified Subcomplex-1, -2 and holocomplex. Insert is shown in enhanced contrast to visualise faint bands from holocomplex. **F**, Exocyst holocomplex was analysed by negative stain and a low-resolution model was generated. The cryo-EM structure of the yeast exocyst (PDB ID 5YFP; ribbons) was placed into the outline (mesh) of this low-resolution shell for visual comparison.

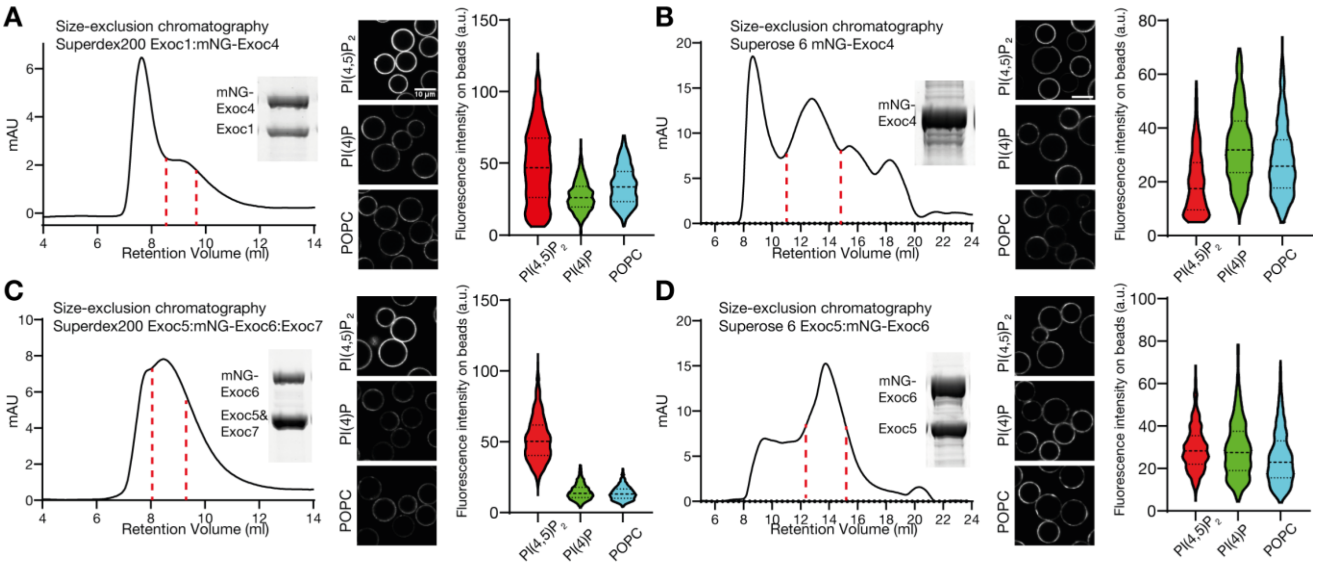

Figure S2. Purification and phosphoinositide binding of exocyst subcomplexes. **Related to Figure 2** **A**, Size-exclusion chromatography of a dimer of Exoc1 with mNeonGreen (mNG)-tagged Exoc4. **B**, Exoc1:4 dimer was added to membrane-coated beads. These were formed from a lipid composition of 84.9% 1-palmitoyl-2-oleoyl-glycero-3-phosphocholine (POPC), 10% phosphatidylserine and 5% phosphoinositide or POPC, doped with 0.1% rhodamine-DPPE. Individual beads were segmented, and mean fluorescent intensity was quantified in ImageJ. n=926-1589 beads. **C**, Size-exclusion chromatography of mNeonGreen (mNG) tagged Exoc4. **D,** Exoc4-mNG was added to membrane-coated beads as in B. n=745-881 beads. **E**, Size-exclusion chromatography of trimer of Exoc5:6:7 with mNeonGreen (mNG)-tagged Exoc6. **F**, Exoc5:6:7 timer was added to membrane-coated beads as in B. n=1032-1239 beads. **G**, Size-exclusion chromatography of dimer of Exoc5:6 with mNeonGreen (mNG) tagged Exoc6. **H**, Exoc5-6 dimer was added to membrane-coated beads as in B. n=1025-1178 beads.

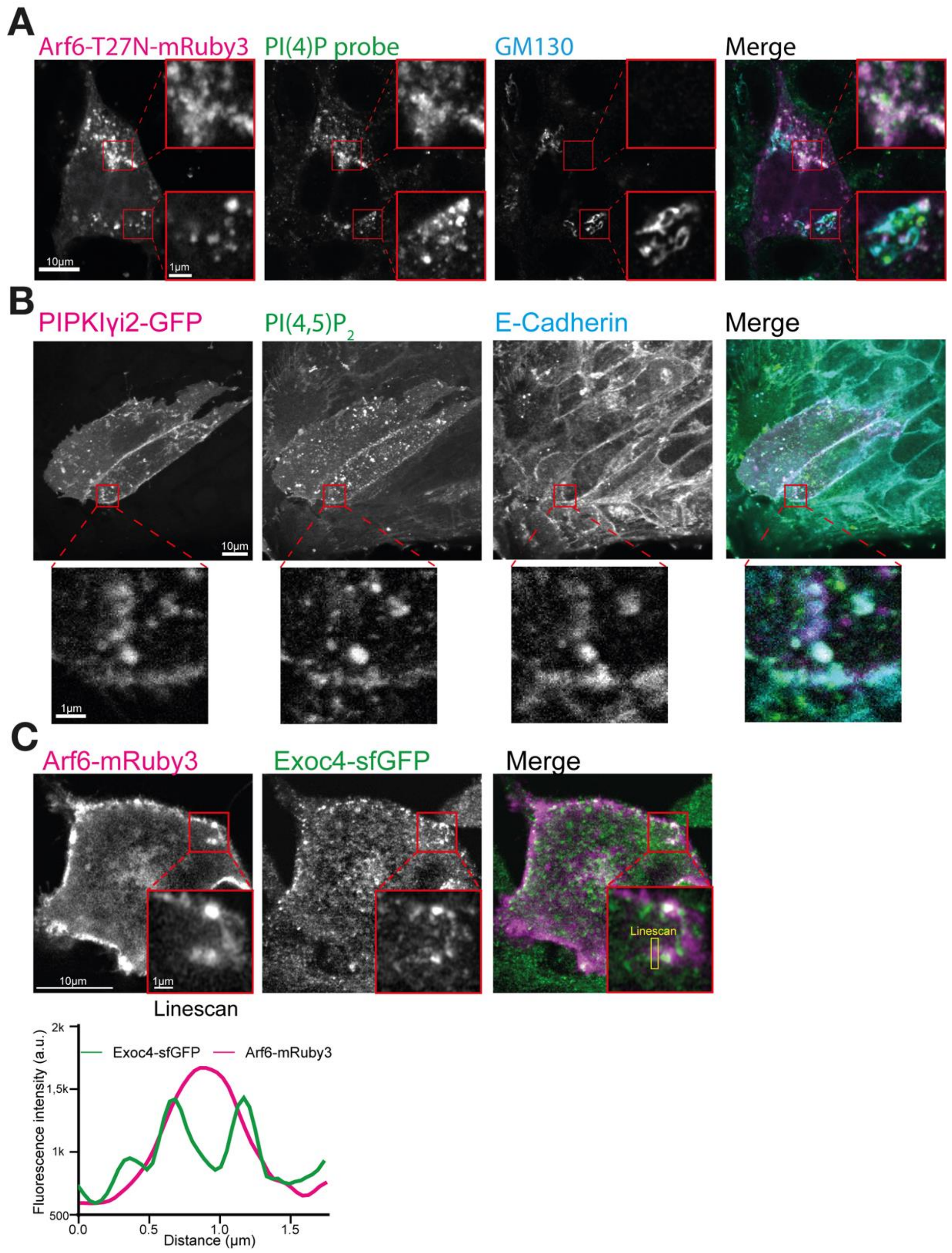

Figure S3. Subcellular localisation of phosphoinositides and Exoc4. Related to Figure 5
A, NMuMG cells were transfected with Arf6-T27N tagged with mRuby3 and stained for PI(4)P using recombinant SidC fused to GFP and conventional antibody staining against GM130. **B**, NMuMG cells were transfected with PIP5K1C tagged with EGFP and stained for PI(4,5)P_2 using recombinant PLCδ-PH fused to RFP, and conventional antibody staining against E-cadherin. **C,** NMuMG cells expressing Exoc4 with endogenous sfGFP tag were transfected with Arf6-WT tagged with mRuby3 and imaged by life cell Airyscan microscopy.
