# Supplementary material for: A mechanism for exocyst-mediated tethering via Arf6 and PIP5K1C-driven phosphoinositide conversion
Source: Curr Biol. 2022 Jul 11;32(13):2821–2833.e6. doi: 10.1016/j.cub.2022.04.089 (PMC9382030; doi:10.1016/j.cub.2022.04.089)

Full Gels

Superdex 200 Exoc1:mNG-Exoc4

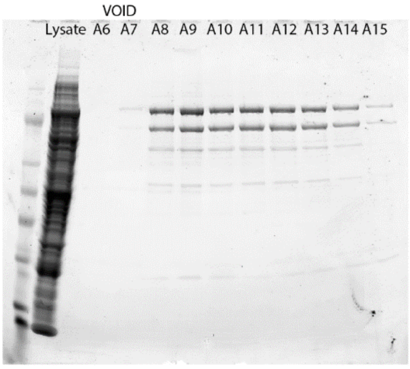

Superdex 200 Exoc5:mNG-Exoc6:Exoc7

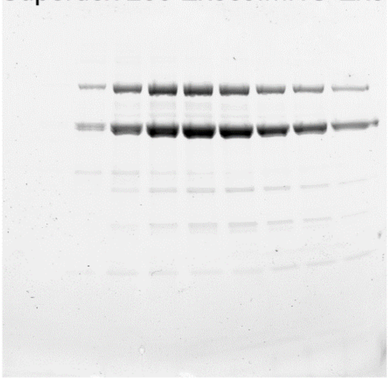

Superose 6 mNG-Exoc4

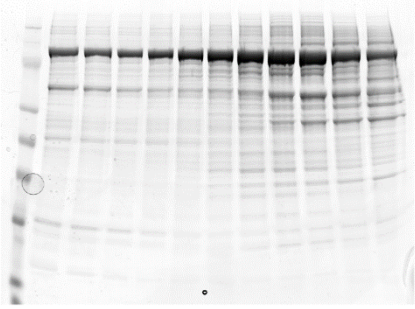

Superose 6 Subcomplex-1

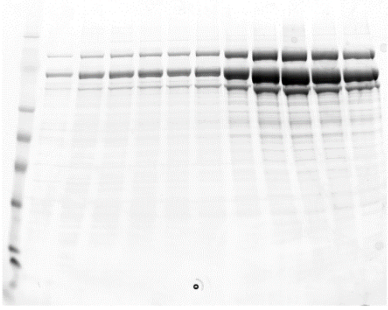

Superose 6 Exoc5:mNG-Exoc6

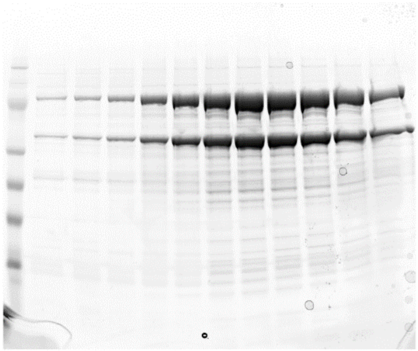

Superose 6 Subcomplex-2

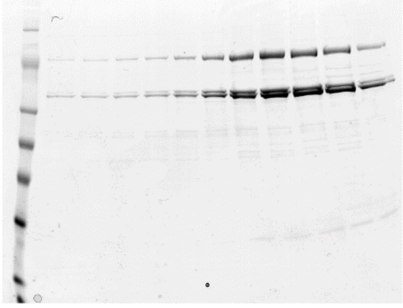

Superose 6 PIP5K1C

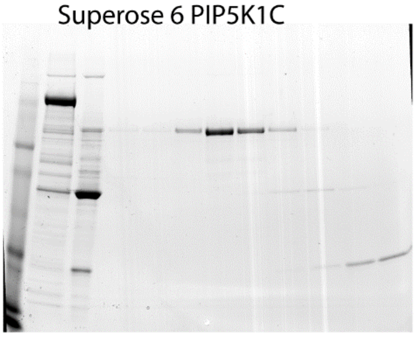

Superose 6 Octamer

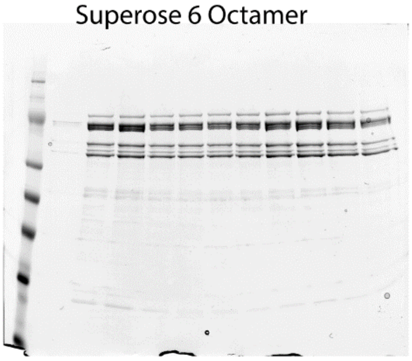

Full gels - interaction screens

Dimeric interaction screen

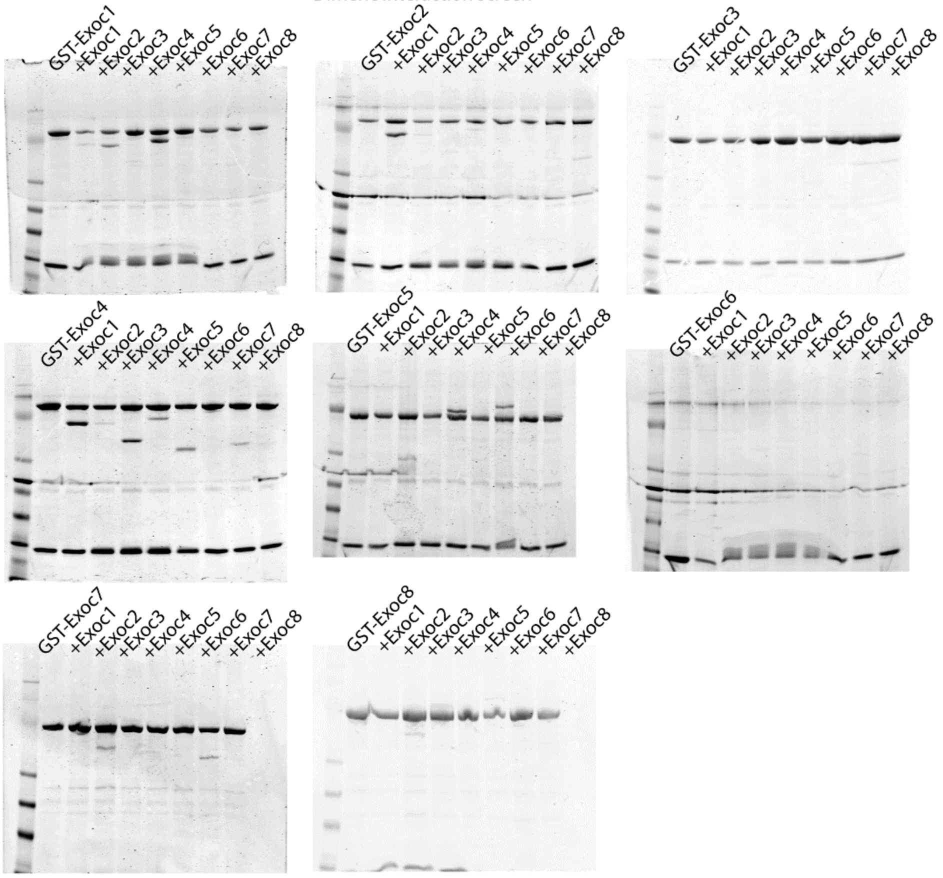

Trimeric interaction screen

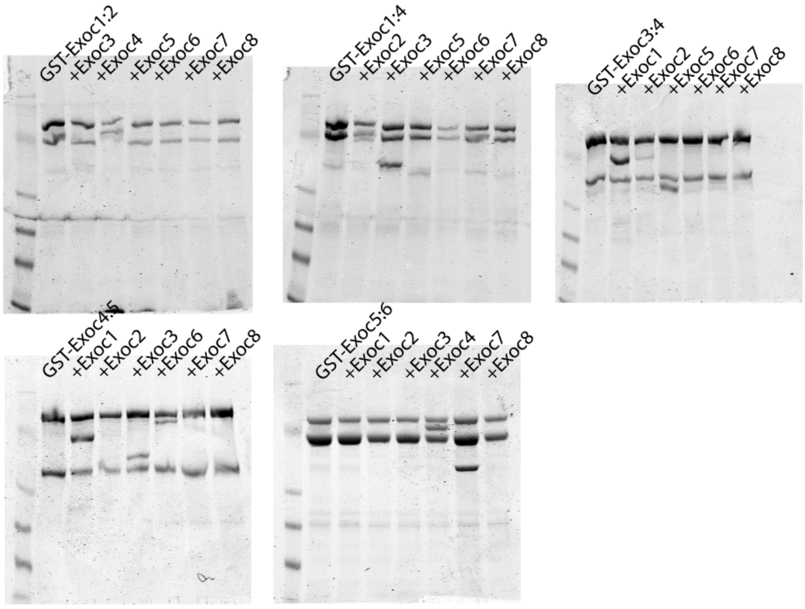

Tetrameric interaction screen

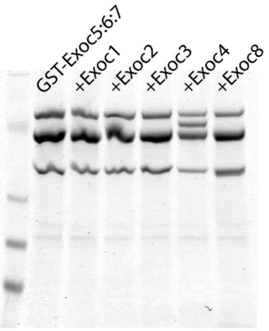

Pentameric interaction screen

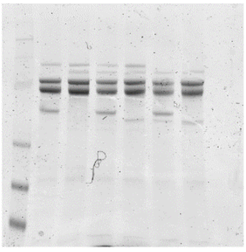

Hexameric interaction screen

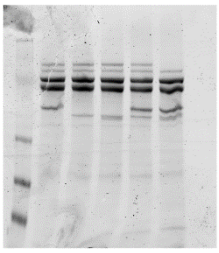

Heptameric interaction screen

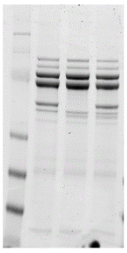

Supplement: Supplementary file 2 [file mmc2.pdf]
