# Supplementary material for: A mechanism for exocyst-mediated tethering via Arf6 and PIP5K1C-driven phosphoinositide conversion
Source: Curr Biol. 2022 Jul 11;32(13):2821–2833.e6. doi: 10.1016/j.cub.2022.04.089 (PMC9382030; doi:10.1016/j.cub.2022.04.089)
Supplement: Supplementary file 3 [file mmc3.pdf]

Current Biology

Article

A mechanism for exocyst-mediated tethering via Arf6 and PIP5K1C-driven phosphoinositide conversion

Graphical abstract

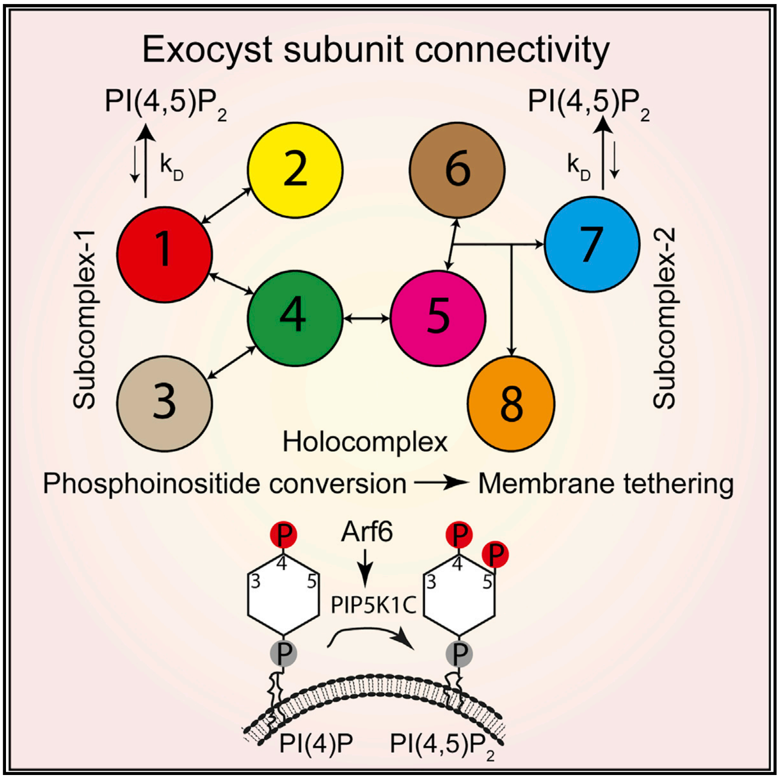

Highlights

- Complete reconstitution and subunit connectivity of the human exocyst complex
- Binding to $PI(4,5)P_2$ in *trans* by each subcomplex enables membrane tethering
- PI(4)P to PI(4,5)P2 conversion is sufficient for exocyst recruitment and tethering
- Arf6 controls phosphoinositide conversion by PIP5K1C in cells and *in vitro*

Authors

Hannes Maib, David H. Murray

Correspondence

dhmurray@dundee.ac.uk

In brief

How are vesicles tethered to the plasma membrane during polarized membrane trafficking? Through complete reconstitution of the mammalian exocyst complex, Maib and Murray show that phosphoinositide conversion of PI(4)P into $PI(4,5)P_2$ by PIP5K1C is controlled by the small GTPase Arf6, and the conversion is minimally sufficient for exocyst-mediated tethering.

Maib & Murray, 2022, Current Biology *32*, 2821–2833
July 11, 2022 © 2022 The Author(s). Published by Elsevier Inc.
https://doi.org/10.1016/j.cub.2022.04.089

Current Biology

CellPress
OPEN ACCESS

Article

A mechanism for exocyst-mediated tethering via Arf6 and PIP5K1C-driven phosphoinositide conversion

Hannes Maib[1] and David H. Murray[1,2,*]
[1]University of Dundee, School of Life Sciences, Division of Cell & Developmental Biology, Dow Street, Dundee DD1 5EH, UK
[2]Lead contact
*Correspondence: dhmurray@dundee.ac.uk
https://doi.org/10.1016/j.cub.2022.04.089

SUMMARY

Polarized trafficking is necessary for the development of eukaryotes and is regulated by a conserved molecular machinery. Late steps of cargo delivery are mediated by the exocyst complex, which integrates lipid and protein components to tether vesicles for plasma membrane fusion. However, the molecular mechanisms of this process are poorly defined. Here, we reconstitute functional octameric human exocyst, demonstrating the basis for holocomplex coalescence and biochemically stable subcomplexes. We determine that each subcomplex independently binds to phosphatidylinositol 4,5-bisphosphate (PI(4,5)P_2), which is minimally sufficient for membrane tethering. Through reconstitution and epithelial cell biology experiments, we show that Arf6-mediated recruitment of the lipid kinase PIP5K1C rapidly converts phosphatidylinositol 4-phosphate (PI(4)P) to PI(4,5)P_2, driving exocyst recruitment and membrane tethering. These results provide a molecular mechanism of exocyst-mediated tethering and a unique functional requirement for phosphoinositide signaling on late-stage vesicles in the vicinity of the plasma membrane.

INTRODUCTION

Eukaryotic cell biology is characterized by directional membrane trafficking pathways supporting membranous organelles. These structures are interconnected, with logistics facilitated by both vesicular transport mechanisms and direct membrane contact sites. Strikingly, the specialization of organelles and their distinct identities are maintained, which are results of continuous sorting and exchange of phospholipid and protein content. In the polarized trafficking pathway, this sorting includes polarity determinants destined to the apical or basal domains of epithelia, integrins, and metalloproteases to the leading edge of invasive cancers and the transport of receptors to post-synaptic densities in neurons.[1,2] The diversity of these cargoes highlights that the molecular mechanisms of polarized trafficking and its regulation are fundamental to a broad swath of cell biology, including organismal development,[3] tissue integrity,[1] and viral egress.[4] Importantly, these distinct cargo carriers with diverse cellular functions are all ultimately trafficked to, and fused with, the plasma membrane in the final step of their journey. However, although the molecular basis for the final step of membrane fusion is well established, a generalizable mechanistic model for the delivery of these diverse cargo carriers remains outstanding.

Orchestration of polarized trafficking requires a highly conserved trafficking machinery. Early screens for factors responsible for polarized secretion in yeast identified the components of the exocyst complex and its regulatory machinery, including small GTPases.[5] The connection of this complex to plasma membrane targeting of cargo vesicles in yeast, plant, and mammalian cells is well established.[6,7] However, models for exocyst-dependent trafficking invoke diverse mechanisms, including GTPase-mediated assembly,[8] interconnections with the SNARE fusion machinery,[9,10] and membrane-associated cellular factors.[11–13] Exocyst-regulatory factors are linked to phosphoinositide signaling, most notably evidenced by Rab11[14] and Arf6.[15–19] Indeed, exocyst conservation[20] and connection of lipid kinases to polarized secretion[21–25] suggest a similar interdependence of phosphoinositides and exocyst function.

Phosphoinositides are a family of phospholipids that are signposts for membrane identity.[26] Their interconversion by lipid kinases and phosphatases is crucial during membrane trafficking and especially well characterized in the endo/lysosomal system.[27] At the plasma membrane, PI(4,5)P_2 is enriched and is important for cargo delivery and fusion.[15,28,29] However, the membrane identity of cargo vesicles directly prior to plasma membrane delivery is poorly defined. Importantly, phosphatidylinositol-4 phosphate is established on vesicles originating from both the Golgi and recycling endosomes.[30–32] Furthermore, the delivery of cell polarity proteins to the plasma membrane, such as ß-integrin and E-cadherin, is dependent on the direct recruitment of phosphatidylinositol 4-phosphate 5-kinase type-1 gamma (PIP5K1C), which converts PI(4)P into PI(4,5)P_2.[25,33] This dependence implies that the phosphoinositide composition of some cargo vesicles is converted to PI(4,5)P_2 along the route to the plasma membrane.[32] Indeed, this outcome has been directly observed during the polarized trafficking of Par proteins

This is an open access article under the CC BY license (http://creativecommons.org/licenses/by/4.0/).

during oogenesis in *Drosophila*.[34] However, no mechanism is known by which PI(4,5)P_2-positive trafficking vesicles are tethered to the plasma membrane.[35]

The exocyst is a multi-subunit tethering complex[2] formed from eight homologous subunits: Exoc1–8 in mammals and Sec3, Sec5, Sec6, Sec8, Sec10, Sec15, Exo70, and Exo84 in yeast. Importantly, two distinct subunits bind to PI(4,5)P_2.[36,37] The binding sites are located at a conserved pleckstrin homology (PH) domain in Exoc1 and in a polybasic region of Exoc7. All exocyst subunits bear a coiled-coil followed by an alpha-helical repeat region.[38] Through coiled-coil assembly, two exocyst heterotetrameric subcomplexes form, with Exoc1–4 as part of subcomplex-1 and Exoc5–8 in subcomplex-2.[38–40] The stability of coiled-coil structures[41] and the *in vivo* colocalization of exocyst subunits[42,43] suggest that the exocyst is present as either tetrameric subcomplexes or a full octamer. Indeed, each subcomplex harbors a distinct PI(4,5)P_2 binding site, although the function of this duplication is unknown.

The interconnectivity of exocyst subunits enables a potential for regulatory modification through disassembly of the holocomplex. Indeed, live-cell imaging of endogenously tagged exocyst subunits suggests that although the subcomplexes are recruited to vesicles independently, they arrive at the plasma membrane together.[42] Furthermore, deletion of one single subunit leads to a concomitant decrease in membrane association[36] and vesicle delivery,[42] highlighting the full holocomplex as the functional unit for vesicle tethering with the plasma membrane. However, a common mechanism for exocyst-mediated tethering of cargo carriers to the plasma membrane is elusive.

Here, we reconstitute the complete human exocyst complex from its individual subunits to address the mechanism by which it tethers vesicles to the plasma membrane during polarized trafficking. We find that the complete octamer readily associates from its separate subunits and is suborganized into two distinct subcomplexes with differing modes of assembly. Furthermore, we show that each subcomplex independently binds to PI(4,5)P_2 and that the holocomplex is able to tether two opposing PI(4,5)P_2-positive membranes. Using cryo-electron tomography, we directly visualize this exocyst-dependent tethering of liposomes and determine a regular tethering distance consistent with the known dimensions of the exocyst complex. Next, we determined how exocyst function is linked to PI(4,5)P_2. *In vitro* reconstitution of phosphoinositide conversion by PIP5K1C is sufficient to drive both exocyst recruitment and membrane tethering. Using endogenous knockin cell lines, we observe exocyst colocalization with PIP5K1C at dynamic subdomains of the plasma membrane. Finally, we demonstrate that PI(4)P to PI(4,5)P_2 conversion is driven by Arf6 activation both *in vitro* and in cells. Therefore, we propose a model in which phosphoinositide conversion, mediated by Arf6 and PIP5K1C, regulates exocyst-dependent membrane tethering.

RESULTS

Complete human exocyst connectivity and reconstitution

To understand the mechanism of exocyst holocomplex assembly and tethering, we reconstituted the human exocyst complex using baculovirus-mediated insect cell expression (Figure 1).[44] We co-expressed every combination of exocyst subunits in pairs, with one subunit harboring a single GST-tag for isolation (Figure 1A). Subsequently, we performed pulldowns of the GST-tagged bait subunit to determine if the prey is directly isolated in this overexpression approach. We used SDS-PAGE followed by Coomassie staining to identify binary interaction partners between the subunits of the exocyst holocomplex (Figure 1B), resulting in several apparent dimers. Next, using the information garnered by the dimer analysis, we co-expressed and analyzed the logical trimers (Figure 1C), determining that Exoc7 binding requires the formation of an initial Exoc5:6 dimer. Subsequently, because we observed no interaction of Exoc8 in the trimeric interaction experiment (Figure 1C), we isolated a trimer of Exoc5:6:7 and confirmed its binding to Exoc8 (Figure 1D). Most importantly, deriving from these results are two subcomplexes, named subcomplex-1 (Exoc1:2:3:4) and subcomplex-2 (Exoc5:6:7:8). These differ in their assembly, with subcomplex-1 based upon binary subunit interactions and subcomplex-2, a hierarchal assembly. Building on the initial formation of an Exoc5:6 dimer, Exoc7 and Exoc8 are subsequently recruited to form tetrameric subcomplex-2.

To assess the assembly of tetramers to octamers, we separately expressed individual subunits and mixed them upon lysis (Figures S1A–S1C). The results from the subsequent pulldowns enabled us to conclude that exocyst assembly does not require co-translation nor input *in vitro* from small GTPases. Moreover, the human exocyst efficiently self-assembles in a hierarchal manner, evolutionarily conserved from yeast (Figure 1E).[38–40,45] The connection between subcomplex-1 and -2 is dependent on a strong interaction between Exoc4 and Exoc5, resulting in the holocomplex assembly.

The principles of assembly for exocyst intermediates and subcomplexes enabled us to discern a purification strategy for intact complexes. For functional analysis, we included fluorescent tags in positions structurally and empirically determined appropriate (Figures 1F–1H).[46] An assembly mechanism driven by the largely bipartite CorEx regions and stabilized by other multivalent interactions[45] enabled us to identify suitable subunits for purification of the holocomplex.[47] Owing to the central position of Exoc5 in exocyst connectivity, we purified holocomplex by an N-terminal MBP-tag on this subunit. We confirmed holocomplex stoichiometry by quantitative mass spectrometry and stability in assembly by native PAGE (Figures S1H and S1I). Moreover, negative stain electron microscopy revealed individual isolated complexes consistent with stable holocomplex assembly, and processing by single-particle methods results in a low-resolution model with dimensions consistent with that derived from cryo-electron microscopy of the yeast complex (Figure S1J). Importantly, these data rule out the formation of aggregated complexes, and we refer to other studies for a more detailed analysis of the exocyst holocomplex by negative stain.[12,39,45,47] Taken together with the rules for its assembly, reconstitution of the human exocyst opens a unique opportunity to gain mechanistic insight into its function.

Two independent PI(4,5)P_2 binding sites enable exocyst membrane tethering

Key to exocyst function is its ability to bind lipids. A late step in delivery of some cargoes to the plasma membrane is the

CellPress
OPEN ACCESS

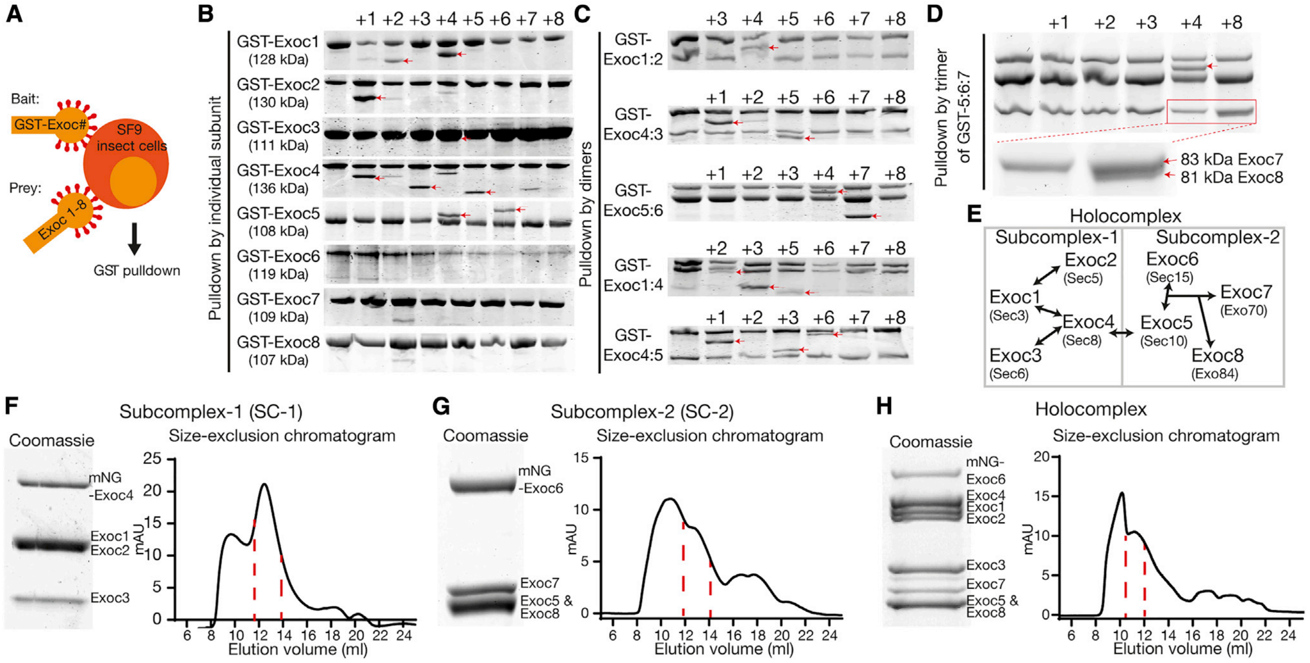

Figure 1. Subunit connectivity and reconstitution of complete human exocyst complex

(A) Schematic of subunit interaction screen. SF9 insect cells were co-infected with baculovirus encoding single GST-tagged subunits of the exocyst complex (bait) together with untagged subunits (prey) and analyzed via GST pulldowns followed by SDS-PAGE and Coomassie staining.

(B and C) Dimeric and trimeric interactions. First lanes are GST-bait without prey. Numbers above gel bands indicate each of the co-expressed exocyst subunits 1–8. Interaction partners are highlighted with red arrowheads.

(D) Tetrameric interactions. GST-Exoc5 together with Exoc6 and Exoc7 were co-expressed with the remaining subunits.

(E) Schematic of complete human exocyst subunit connectivity. Holocomplex is divided into subcomplex-1 and subcomplex-2, which are joined by an interaction of Exoc4 with Exoc5. Names of yeast subunits are given for reference.

(F–H) Biochemical reconstitution of exocyst subcomplex-1 and -2 and holocomplex with size-exclusion chromatograms (Superose 6 Increase 10/300 GL; void volume, 9 mL). Both subcomplexes-1 and 2 elute at a retention volume of 13 mL, whereas full holocomplex elutes at 11 mL. Coomassie-stained insets are taken from peak fractions. Full gels are found in Data S1.

See also Figure S1.

conversion of a subset of vesicular phosphoinositides to PI(4)P.[30,31] This change of lipid chemistry is concomitant with trafficking of vesicles destined for the plasma membrane that harbor cargoes critical for polarity maintenance and cellular homeostasis.[34,35,48,49] Exoc1 and Exoc7 bind phosphoinositides in isolation,[36,37] but cooperativity or independence in membrane binding has not been assessed.

To determine the selectivity of exocyst complexes to phosphoinositides, we measured the binding of purified, fluorescently tagged holo- and sub-complexes for PI(4)P or PI(4,5)P_2 in membranes chosen to mimic the plasma membrane. We formed supported-lipid bilayers on silica beads with liposomes composed of 85% 1-palmitoyl-2-oleoyl-glycero-3-phosphocholine (POPC), 10% phosphatidylserine, and 5% phosphoinositide (Figure 2A).[50,51] By including N-terminal mNeonGreen (mNG) in Exoc4 for subcomplex-1 and Exoc6 for both subcomplex-2 and the holocomplex, we observed recruitment of the complexes to membrane-coated beads by confocal microscopy (Figure 2B). To statistically compare exocyst recruitment, we segmented and quantified the integral fluorescence intensity of the bound exocyst per membrane-coated bead (Figure 2C) (POPC/PI(4)P versus PI(4,5)P_2; $p < 0.01$). The exocyst and Exoc1/Exoc7-containing subcomplexes are unambiguously recruited selectively to PI(4,5)P_2 and not PI(4)P-containing membranes at non-saturating conditions (100 nM complex). Moreover, by harnessing the assembly hierarchy to generate exocyst subcomplexes, we explicitly demonstrate the requirement for Exoc1 or Exoc7 subunits for membrane binding (Figure S2).[35] Strikingly, the novel observation that both subcomplex-1 and -2 independently bind PI(4,5)P_2, and not PI(4)P, questions the potential role of avidity in membrane binding by the holocomplex.

To quantitate the independence and modality of lipid binding, we determined the relative affinities of subcomplexes-1, -2, and the holocomplex for PI(4,5)P_2 membranes. We adapted the membrane-coated bead assay with varying protein concentrations to obtain their relative affinities for PI(4,5)P_2. By systematic imaging and analysis, we determined that both subcomplexes-1 and -2 were recruited to membranes with similar strength (subcomplex-1, $K_{D,relative}$ = 36.1 nM; subcomplex-2, $K_{D,relative}$ = 38.5 nM) (Figures 2D and 2E). However, exocyst holocomplex binding to membranes of identical composition is significantly weaker ($K_{D,relative}$ = 99.4 nM; AIC < 0.01%) (Figure 2F). Interestingly, this result makes monovalent binding to PI(4,5)P_2 by the exocyst plausible, although with an increased kinetic off rate compared with individual subcomplexes. Moreover, it negates

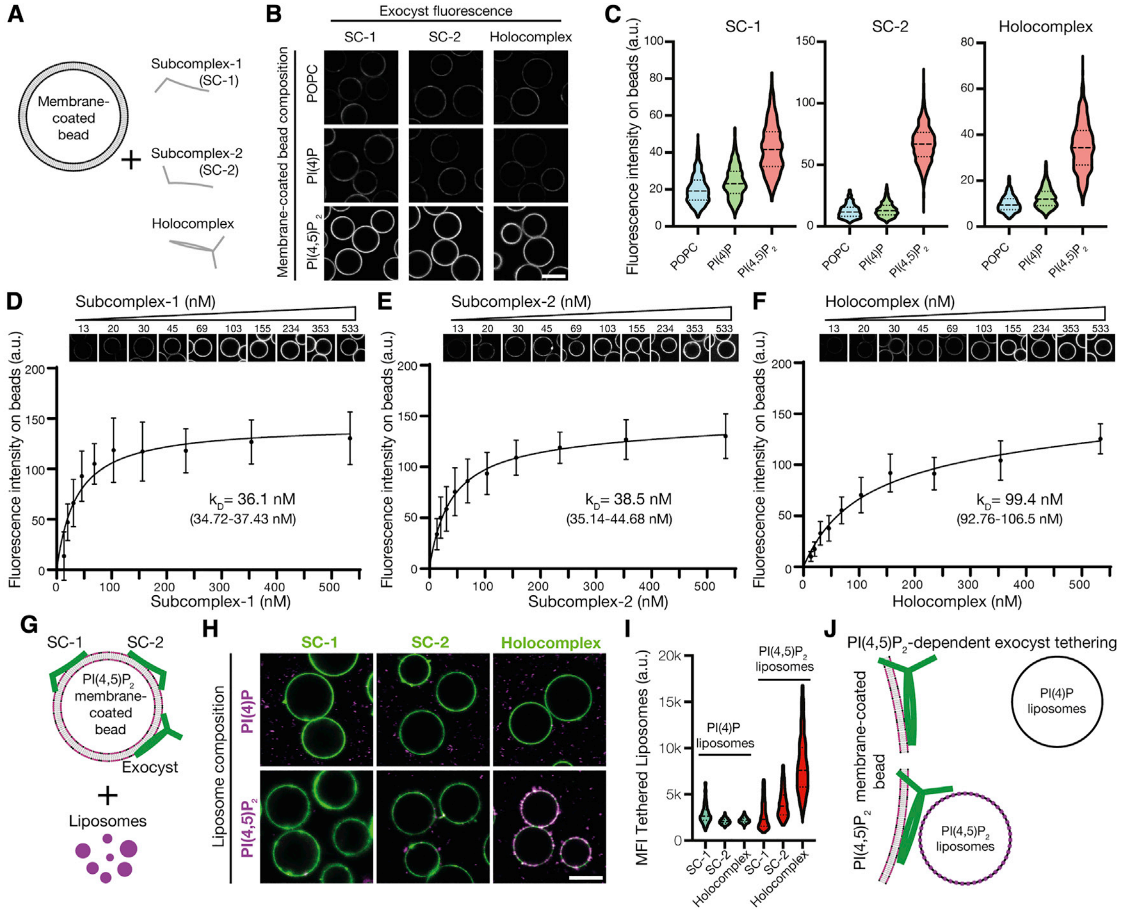

Figure 2. Independent binding of exocyst subcomplexes-1 and -2 to PI(4,5)P_2 enables holocomplex tethering

(A) Schematic of phosphoinositide specificity experiment. Membrane-coated beads of distinct lipid composition were generated. To these beads, fluorescently tagged exocyst subcomplexes were added to assess membrane binding by fluorescence microscopy.

(B) Reconstituted fluorescently tagged subcomplexes-1 and -2 or holocomplex were added at 100 nM to membrane-coated beads. These were formed from a lipid composition of 84.9% 1-palmitoyl-2-oleoyl-glycero-3-phosphocholine (POPC), 10% phosphatidylserine, and 5% phosphoinositide or POPC, doped with 0.1% rhodamine-DPPE. Beads were imaged by confocal microscopy. Scale bar, 10 μm.

(C) Membrane-coated beads were segmented using the rhodamine lipid signal as a membrane mask, and the mean fluorescence intensities of exocyst complexes bound to each bead were determined. Violin plots, 877–1,395 segmented beads per condition.

(D–F) Relative PI(4,5)P_2 membrane binding affinities for the exocyst complexes. Subcomplexes-1 and -2 or holocomplex was added in a dilution series to membrane-coated beads containing 5% PI(4,5)P_2 and imaged by confocal microscopy. Individual beads were segmented, and a binding saturation curve was fitted resulting in mean kD determination with 95% confidence intervals given. In total, 340–1,359 beads were quantified per concentration, mean ± standard deviation.

(G) Schematic of liposome tethering experiment. Subcomplexes-1 and -2 or holocomplex (150 nM) were bound to 5% PI(4,5)P_2 membranes. Liposomes doped with Atto647N-DOPE and composed with either 5% PI(4)P or PI(4,5)P_2 were added.

(H and I) Membranes and exocyst complexes were imaged by confocal microscopy, segmented, and the fluorescent intensity of tethered liposomes was measured. Violin plots from 430–778 individual beads. MFI, mean fluorescent intensity. Scale bar, 10 μm.

(J) Exocyst holocomplex tethers two PI(4,5)P_2, but not PI(4)P, containing membranes to each other.

See also Figure S2.

the possibility of avidity in lipid-mediated recruitment of the holocomplex to membranes and further suggests that conformational changes within the holocomplex could account for its decreased affinity toward PI(4,5)P_2. These results, together with phosphoinositide selectivity (Figures 2B, 2C, and S2) and the holocomplex assembly mechanics (Figure 1), suggest that exocyst lipid binding occurs in a *trans* configuration. Therefore, we hypothesized that the holocomplex is capable of tethering two distinct PI(4,5)P_2-containing membranes.

To test this hypothesis, we recruited the fluorescently tagged exocyst or subcomplexes (150 nM) to PI(4,5)P_2-containing POPC membrane-coated beads (Figure 2G), as previously demonstrated (Figure 2B). Next, we added fluorescently labeled liposomes harboring either PI(4)P or PI(4,5)P_2 and quantified the fluorescence intensity of tethered liposomes.[50] Membranes were only tethered in the presence of the exocyst holocomplex when both contained PI(4,5)P_2 (Figure 2H). Individual exocyst subcomplexes failed to tether liposomes, and in the absence

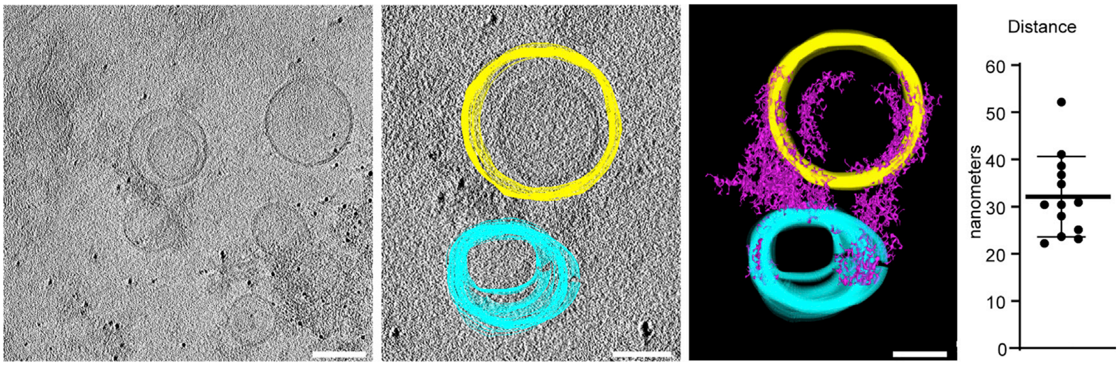

Figure 3. Cryo-electron tomography of exocyst-mediated liposome tethering
Purified exocyst holocomplex was added to 5% PI(4,5)P_2 containing liposomes in the presence of 5 nm protein-A coated gold fiducial markers, plunge frozen in liquid ethane and tilt series were acquired. Tomograms were reconstructed resulting in a three-dimensional data set, and liposomes segmented manually through the z axis slices (yellow and cyan). Exocyst holocomplex, present as proteinaceous material bound to the liposomes, was segmented using auto-thresholding (purple), which also partially delineates electron-dense portions of the liposomes. A model was generated from this segmentation, and the distance of closest approach between tethered liposome pairs was determined (mean ± standard deviation). Scale bars, 100 nm.

of liposomal PI(4,5)P_2, we observed no statistically significant tethering (Figure 2I). These data provide *in vitro* evidence for a minimal mechanism of exocyst-mediated membrane tethering (Figure 2J).

To verify these findings and to directly observe the exocyst holocomplex on membranes, we harnessed cryo-electron tomography. We prepared samples of 5% PI(4,5)P_2-containing POPC liposomes extruded to 100 nm and mixed with exocyst. Samples were plunge frozen, micrographs acquired, and tomograms reconstructed to visualize the samples in three dimensions, including both lipid membranes and proteinaceous material corresponding to the exocyst holocomplex. In tomograms, we observed protein density, dispersed in regions devoid of liposomes. In regions with liposomes, we observed protein density on the membrane and tethering (Figure 3). By manual segmentation of the tomograms in three dimensions, we noted and quantified a regular distance between pairs of membranes at the position of closest approach and bridged by protein density. This resulted in an average distance of 32.1 nm, consistent with the dimensions of the exocyst complex from yeast,[38] providing additional evidence for exocyst-mediated tethering of PI(4,5)P_2 membranes.

Phosphoinositide conversion drives membrane tethering

In polarized trafficking, phosphoinositide dynamics are required for delivery of vesicles prior to the engagement of fusion machinery. A key factor in these dynamics is the endosomal recruitment of the phosphatidylinositol-5 kinase PIP5K1C.[18,30,33] To determine if phosphoinositide conversion drives exocyst-mediated tethering, we reconstituted human PIP5K1C to convert PI(4)P into PI(4,5)P_2 in a modified assay for exocyst recruitment (Figure 4A).[52] We mixed recombinant fluorescently tagged exocyst (100 nM) with PI(4)P-positive membrane-coated beads (as in Figure 2B). After the addition of reconstituted PIP5K1C (150 nM), we observed exocyst recruitment to membranes in an ATP-dependent manner by confocal fluorescence microscopy (Figure 4B). In this system, limited by mixing, the conversion and subsequent exocyst recruitment occurred rapidly once underway. This recruitment is consistent with the observed affinities of the exocyst to PI(4,5)P_2 membranes (Figures 2D–2F) and provides a baseline for the activity of PIP5K1C, in the absence of a mechanism for lipid kinase recruitment. Critically, phosphoinositide conversion not only drove exocyst membrane recruitment but also drove tethering. We mixed PI(4,5)P_2-positive liposomes, the exocyst, and PIP5K1C together with PI(4)P-positive membrane-coated beads. Following conversion until a saturating time point, we evaluated the fluorescence intensity of tethered liposomes (Figures 4C and 4D). We observed a clear dependence of liposome tethering on phosphoinositide conversion and a linear dependence on the amount of exocyst recruited. Therefore, *de novo* generation of PI(4,5)P_2 by PIP5K1C mediates *in vitro* exocyst-dependent tethering. This suggests that late-stage phosphoinositide conversion, on some vesicles destined to the plasma membrane, could drive tethering during polarized trafficking.

To validate these findings in a cellular system, we used genome edited knockin cells from mouse mammary epithelia (NMuMG) with sfGFP at the C terminus of Exoc4.[42] These cell lines have previously been described, and the fluorescent tag does not affect exocyst function. Importantly, these cells readily differentiate into an epithelial sheet with apicobasal polarity and are therefore well suited to observe the exocyst during polarized trafficking. Using live-cell Airyscan microscopy,[53] we determined that the exocyst is mainly located at basolateral and junctional membranes. Here, the exocyst is organized into dynamic subdomains that are stable for several minutes (Figure 4E). This localization is consistent with its role as a tethering complex in the direct vicinity of the plasma membrane.[2,42] To address the link between phosphoinositide conversion and exocyst function, we exogenously expressed PIP5K1C-mScarlet-I in these cell lines and performed live-cell imaging (Figure 4F). We observed PIP5K1C and Exoc4-sfGFP together at dynamic subdomains in the direct vicinity of the plasma membrane. These data provide further evidence for a functional link between the exocyst and the generation of PI(4,5)P_2.[33]

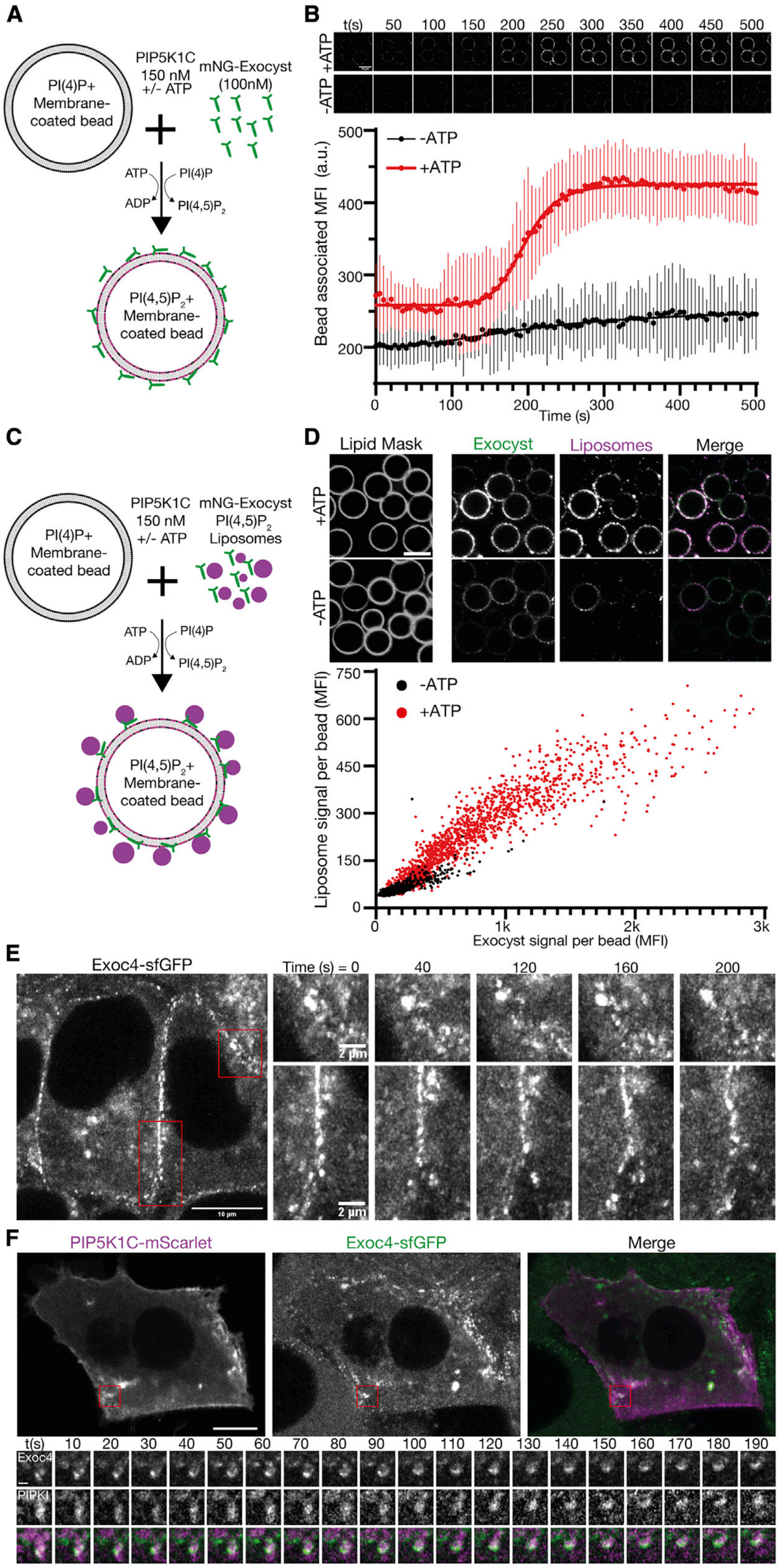

Figure 4. Phosphoinositide conversion drives exocyst-mediated membrane tethering

(A) Schematic of exocyst recruitment by phosphoinositide conversion. Membrane-coated beads containing PI(4)P were mixed with exocyst (100 nM) and PIP5K1C (150 nM) in the presence and absence of ATP.

(B) Exocyst is recruited to membrane-coated beads upon ATP-dependent PI(4)P to PI(4,5)P_2 conversion, and imaged by confocal microscopy. Beads were segmented, and the associated exocyst mean fluorescent intensity was measured over time. Mean ± standard deviation, n = 44, 68 beads. Scale bar, 10 μm.

(C) Schematic of membrane tethering by phosphoinositide conversion. PI(4,5)P_2 containing liposomes were added to the phosphoinositide conversion experiment.

(D) The intensity of bound exocyst and tethered liposomes was determined after 30 min, and the mean fluorescent intensity of each was plotted per bead. n = 1,440, 1,864 beads for −ATP and +ATP, respectively. Scale bar, 10 μm.

(E) NMuMG cells with endogenous Exoc4-sfGFP were imaged by live-cell Airyscan microscopy. Insets show the dynamics of plasma membrane-associated exocyst subdomains.

(F) Cells were transfected with mScarlet-tagged PIP5K1C and imaged by live-cell Airyscan microscopy. Inset shows time series of exocyst colocalization with PIP5K1C at dynamic subdomains of the plasma membrane. Scale bar, 10 μm, inset scale bar, 1 μm.

CellPress
OPEN ACCESS

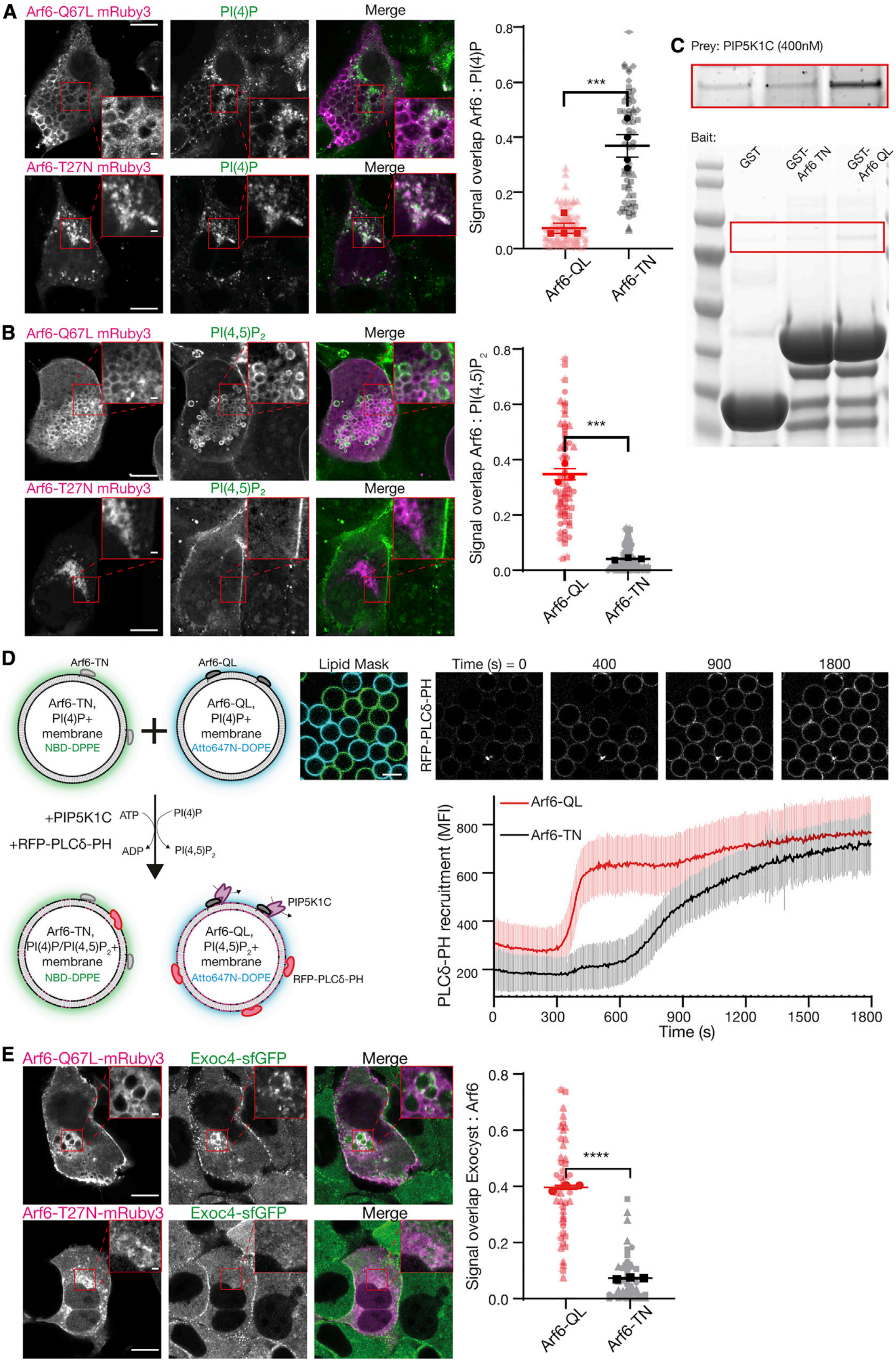

(legend on next page)

Arf6 mediates PIP5K1C-dependent $PI(4,5)P_2$ generation and exocyst recruitment

The conversion of PI(4)P into $PI(4,5)P_2$ at intracellular membranes is catalyzed by the Arf6-mediated recruitment of PIP5K1C,[25] which is in part also dependent on the presence of anionic lipids.[52] We hypothesized that regulation of Arf6, by virtue of recruiting PIP5K1C, drives $PI(4,5)P_2$ generation and subsequent exocyst-mediated tethering. To observe cellular phosphoinositides, we harnessed a staining approach using purified phosphoinositide-binding probes, allowing us to visualize lipid populations in fixed cells (Figures S3A and S3B).[54] Briefly, recombinant purified SidC,[55] and PLCδ-PH domain,[56] tagged with GFP were used as probes in fixed and permeabilized NMuMG cell lines to visualize PI(4)P and $PI(4,5)P_2$, respectively. We observed strong colocalization of immunostained Golgi-resident GM130 with PI(4)P by SidC staining (Figures S3A and S3B). Respectively, for PLCδ-PH staining, we observed plasma membrane alongside intracellular vesicles harboring $PI(4,5)P_2$ and E-cadherin in PIP5K1C expression conditions (Figure S3B). This methodology allows us to robustly detect phosphoinositides in cells while avoiding overexpression artifacts of lipid probes.

To address Arf6-mediated regulation of the conversion of PI(4)P to $PI(4,5)P_2$, we used expression of constitutively active or inactive Arf6 to disrupt GTPase cycling (Arf6-Q67L and Arf6-T27N, respectively).[57] Expression of Arf6-T27N results in the well-described phenotypes of post-Golgi halted secretion, whereas expression of Arf6-Q67L led to a progressive accumulation of plasma membrane-derived vesicles resulting from overactive macropinocytosis (e.g.,Figure 5A [top left panel]).[15,58,59] We took advantage of these phenotypes to determine the localization of phosphoinositides to the resulting internal membranes. Arf6-T27N positive membranes contained PI(4)P (Figure 5A) but not $PI(4,5)P_2$ (Figure 5B). Moreover, they were only partially positive for a Golgi marker (Figure S3A [top insets]), indicating an intracellular PI(4)P pool not associated with the Golgi. In contrast, membranes positive for Arf6-Q67L were strongly enriched with $PI(4,5)P_2$ (Figure 5B), but not PI(4)P (Figure 5A). The observed differences in colocalization of phosphoinositides with Arf6 correlates directly with its activation state and is consistent with a direct role in the generation of $PI(4,5)P_2$.

To evaluate the mechanism by which Arf6 activation leads to the accumulation of $PI(4,5)P_2$, we directly assessed PIP5K1C binding to Arf6.[16,60] Purified PIP5K1C interacts with purified Arf6 in a GTPase activity-dependent manner (Figure 5C), consistent with results from cell biological experiments[15,60] and demonstrating that PIP5K1C is an effector of Arf6.[16] This result explains the elevated levels of $PI(4,5)P_2$ on intracellular membranes in Arf6-Q67L expression (Figure 5B). Moreover, the direct interaction led us to hypothesize that Arf6-mediated lipid kinase recruitment drives rapid phosphoinositide conversion.

To directly evaluate the biochemical characteristics of Arf6-mediated phosphoinositide conversion in a reduced system, we reconstituted the lipid kinase and membrane-coated beads harboring PI(4)P together with Arf6. We conjugated either Arf6-T27N or Arf6-Q67L to membrane-coated beads harboring the high-affinity TrisNTA-DODA lipid via N-terminal 6xHis tags.[61] Both populations were distinctly doped with fluorescent lipids and observed simultaneously by microscopy (Figure 5D). After addition of PIP5K1C at kinetic concentrations (12.5 nM) to these membranes, we observed the production of $PI(4,5)P_2$, assessed by the recruitment of RFP-PLCδ-PH. As expected, we observed relatively slow recruitment of this probe to Arf6-T27N membranes, reflecting basal activity of the lipid kinase (Figure 5E). In contrast, membranes harboring Arf6-Q67L displayed extremely rapid and cooperative phosphoinositide conversion. This result supports a mechanism, whereby active Arf6 potentiates lipid kinase activity by the direct recruitment of PIP5K1C to the membrane. The phosphoinositide conversion thereby functions as a biochemical switch,[62,63] rapidly redefining the identity of the membrane. We demonstrated that this change of identity is sufficient for exocyst recruitment and tethering (Figure 4), which suggests a key role for phosphoinositide conversion in polarized trafficking.

To directly test this mechanism in a cellular system, we determined the exocyst localization in polarized NMuMG cells expressing Arf6 variants including wild type.[42] In cells expressing the inactive Arf6-T27N, we observed no change to exocyst localization (Figure 5E; compare Figure 4E). In contrast and in addition to the normal exocyst localization, expression of active Arf6-Q67L resulted in significant colocalization with the exocyst (Figure 5E), retargeting the complex to $PI(4,5)P_2$-positive internal membranes. This result demonstrates that the exocyst is linked to Arf6 in an activity-dependent manner. Furthermore, we observe the exocyst at Arf6-WT containing vesicles in the direct vicinity of the plasma membrane (Figure S3C), providing further evidence that octameric exocyst complex assembles on vesicles at or near the plasma membrane to mediate their tethering. Therefore, these experiments support a model in

Figure 5. Arf6 controls $PI(4,5)P_2$ conversion and exocyst recruitment

(A and B) NMuMG cells were transfected with Arf6-Q67L or Arf6-T27N tagged with mRuby3, and subsequently fixed, permeabilized, and stained against either PI(4)P or $PI(4,5)P_2$ using purified SidC or PLCδ-PH fused to GFP, respectively. Signal overlap between channels was determined using the ImageJ plugin Squassh. Plot shows biological repeats (in bold) with individual cells (in background); n = 3–4 repeats with 29–36 individual cells each. Asterisks denote $p < 0.001$. Scale bar, 10 μm; inset, 1 μm.

(C) Purified GST, GST-Arf6-T27N or GST-Arf6-Q67L were bound to resin as bait, and purified PIP5K1C (400 nM) used as prey. Inset (top) is increased contrast.

(D) Membrane-coated beads harboring PI(4)P and either recombinant Arf6-T27N or Arf6-Q67L were distinctly labeled with NBD-DPPE or Atto647N-DOPE and mixed. PIP5K1C (12.5 nM) was added, and the recruitment of purified PLCδ-PH fused to RFP was measured over time. Beads were segmented using either the NBD or Atto647N signal as masks. Plot is mean fluorescent intensity of recruited PLCδ-PH ± standard deviation, quantified in ImageJ. n = 92–99 beads. Scale bar, 10 μm.

(E) NMuMG cells expressing endogenously sfGFP-tagged Exoc4 were transfected with Arf6-Q67L or Arf6-T27N fused to mRuby3 and imaged using Airyscan confocal microscopy. Signal overlap between channels was determined using the ImageJ plugin Squassh. n = 3 repeats with 7–25 individual cells each; asterisks denote $p < 0.001$. Scale bar, 10 μm; inset, 1 μm.

See also Figure S3.

CellPress
OPEN ACCESS

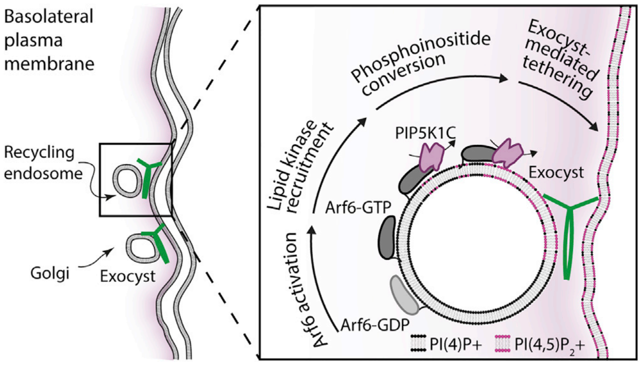

Figure 6. Exocyst-mediated lipid kinase driven membrane tethering
Polarized trafficking vesicles originating either from recycling endosomes or the Golgi are trafficked to the vicinity of the basolateral plasma membrane. Close to the plasma membrane, Arf6 is activated, resulting in recruitment of the PIP5K1C lipid kinase. This kinase converts vesicular PI(4)P into $PI(4,5)P_2$, which results in exocyst recruitment and cargo carrier tethering to the plasma membrane.

which late-stage phosphoinositide conversion is critical to exocyst-mediated tethering in polarized trafficking (Figure 6).

DISCUSSION

Our data support a model whereby exocyst-mediated selectivity and tethering results in part from the coordination of intracellular $PI(4,5)P_2$ conversion at late stages of vesicle delivery to the plasma membrane. We have demonstrated in a reconstitution-based approach the constitutive nature of the exocyst complex and provided new mechanistic evidence for holocomplex-driven tethering. We have identified the role of phosphoinositide binding, and upstream lipid conversion, in regulating functional tethering by the exocyst complex. This lipid kinase-mediated mechanism provides a novel framework for understanding exocyst-dependent delivery during polarized trafficking. The observation that the exocyst holocomplex has two independent membrane binding interfaces, and its ability to tether two $PI(4,5)P_2$-containing membranes, opens up a myriad of regulatory mechanisms to control vesicle tethering *in vivo*. Indeed, other late-stage vesicle delivery events may be independent of phosphoinositide conversion. In such events, it is possible that the exocyst complex binds to $PI(4,5)P_2$ with one of its binding interfaces at the plasma membrane, while being targeted to the vesicle through other mechanisms, such as GTPase or SNARE binding.[8,12,29,64] Importantly, our findings highlight exocyst versatility and the potential for modularity in mediating the delivery of diverse vesicles, carrying equally as diverse cargoes to the plasma membrane.

The direct observation that the exocyst holocomplex tethers two distinct $PI(4,5)P_2$ membranes to each other suggests a physical opposition of the two lipid binding regions of the holocomplex. However, in current structures of the yeast exocyst complex,[38,45] the localization of the lipid binding PH domain of Sec3 (Exoc1) cannot be directly observed, owing to a large flexible linker connecting this domain to the C-terminal portion of the protein and thereby the complex. Mammalian Exoc1 lacks this linker, potentially resulting in a more fixed and distinct location of the PH domain within the holocomplex. Indeed, the two $PI(4,5)P_2$ binding regions of the yeast exocyst may be redundant, as vesicle tethering still occurs if either of them is deleted.[36,37] In contrast, the mammalian exocyst requires both $PI(4,5)P_2$ binding regions for the polarized trafficking of E-cadherin to cellular junctions[33] and the delivery of post-Golgi secretory vesicles to the plasma membrane.[35] An additional layer of complexity arises from the potential for cargo proteins themselves to directly recruit PIP5K1C to trafficking vesicles.[33]

The generation of minor pools of $PI(4,5)P_2$ on intracellular trafficking vesicles must be tightly regulated and places a requirement for the generation of $PI(4,5)P_2$ downstream of endosome exit at the strictest of vicinity to the plasma membrane. Indeed, $PI(4,5)P_2$ is present on internal membranes regulating distinct trafficking events.[65] The most parsimonious model for these observations requires control over the localization of the lipid kinase, whose activity is directly linked to the activation of Arf6.[15] This model mechanistically implicates the activity of Arf6 on vesicles in both recycling[48] and potentially secretory pathways[35,66]; however, its link to plasma membrane delivery at late steps has been unclear. Our data uncover the direct role of Arf6-regulated $PI(4,5)P_2$ generation and suggest a holistic model for exocyst-mediated tethering in polarized trafficking.

Indeed, further layers of regulatory complexity influence the composition of the exocyst holocomplex. The independent $PI(4,5)P_2$ binding of each exocyst subcomplex is paralleled by Ral GTPase-mediated regulation, exerted through Ral binding independently to both Exoc2 in subcomplex-1 and Exoc8 in subcomplex-2.[8] This duality is likely due to an evolutionary duplication event.[20,67] Importantly, the holocomplex is the functional unit required for vesicle tethering with the plasma membrane, and the majority of exocyst appears to be in this state.[42] However, the role of separate subcomplexes is less well understood. Subcomplexes of the exocyst may have a critical role in autophagosome formation and mTOR signaling,[68–70] and their independence in $PI(4,5)P_2$ binding may be specifically relevant to these processes. Furthermore, the complete assembly model of the human exocyst and its separate subcomplexes provide new insights into regulatory pathways such as ULK1- and TBK1-mediated phosphorylation of Exoc7 and Exoc8, respectively.[8,71,72] Our results highlight that these subunits are most capable of disassociation from the holocomplex, which although contrasting with some earlier suggestions,[73] is supported by the hierarchal nature of the human exocyst.[40,42,47] Additionally, the complete model of exocyst subunit interconnection enables cellular mechanisms by which stepwise association of distinct subunits may be regulated, ultimately leading to the formation of holocomplex.

Finally, the role of small GTPases in exocyst-dependent trafficking remains to be clarified. In particular, the activation and interconnections of Rab-family GTPases to Arf6[66,74–76] are enticing in the context of the final steps of cargo carrier delivery to the plasma membrane. The model we present for exocyst-mediated tethering provides a framework for linking the roles of the SNARE membrane fusion machinery, small GTPases, and the myriad of other exocyst binding partners to polarized trafficking.

STAR★METHODS

Detailed methods are provided in the online version of this paper and include the following:

- KEY RESOURCES TABLE
- RESOURCE AVAILABILITY
  - Lead contact
  - Materials availability
  - Data and code availability
- EXPERIMENTAL MODEL AND SUBJECT DETAILS
- METHOD DETAILS
  - Plasmid construction
  - Baculovirus production
  - Exocyst subunit interaction screen
  - Protein purifications
  - Negative stain electron microscopy
  - Lipids, membrane-coated beads, and lipid binding experiments
  - Phosphoinositide conversion assays
  - Cryo-electron tomography
  - Live-cell Airyscan imaging
  - Phosphoinositide staining in cells
  - Arf6 - PIP5K pulldown
- QUANTIFICATION AND STATISTICAL ANALYSIS

SUPPLEMENTAL INFORMATION

Supplemental information can be found online at https://doi.org/10.1016/j.cub.2022.04.089.

ACKNOWLEDGMENTS

We are very grateful to Ian Macara and Mukhtar Ahmed for sharing their cell lines. We thank Hayley Shaw, Aliona Bogdanova, the Evzen Boura lab, and the Jakub Piehler lab for reagents. We acknowledge Ramasubramaniam Sundaramoorthy for EM assistance. We thank Jens Januschke, Jason King, and Kees Weijer for their comments on the manuscript. We gratefully acknowledge the Dundee Imaging Facility, University of Dundee for their support in this work. We acknowledge the Scottish Centre for Macromolecular Imaging (SCMI) and James Streetley for assistance with cryo-EM experiments and access to instrumentation, funded by the MRC (MC_PC_17135) and SFC (H17007). We would like to acknowledge the FingerPrints Proteomics Facility at the University of Dundee, which is supported by the "Wellcome Trust Technology Platform" award (097945/B/11/Z). This work was supported by the Wellcome Trust grant 211193/Z/18/Z to D.H.M. and Royal Society RGS\R2\180284 to D.H.M.

AUTHOR CONTRIBUTIONS

Investigation, H.M.; conceptualization and writing, H.M. and D.H.M.; funding acquisition and supervision, D.H.M.

DECLARATION OF INTERESTS

The authors declare no competing interests.

Received: November 16, 2021
Revised: March 8, 2022
Accepted: April 28, 2022
Published: May 23, 2022

REFERENCES

1. Bryant, D.M., and Mostov, K.E. (2008). From cells to organs: building polarized tissue. Nat. Rev. Mol. Cell Biol. *9*, 887–901. https://doi.org/10.1038/nrm2523.
2. Wu, B., and Guo, W. (2015). The exocyst at a glance. J. Cell Sci. *128*, 2957–2964. https://doi.org/10.1242/jcs.156398.
3. Lecuit, T., and Pilot, F. (2003). Developmental control of cell morphogenesis: a focus on membrane growth. Nat. Cell Biol. *5*, 103–108. https://doi.org/10.1038/ncb0203-103.
4. Wang, R., Simoneau, C.R., Kulsuptrakul, J., Bouhaddou, M., Travisano, K.A., Hayashi, J.M., Carlson-Stevermer, J., Zengel, J.R., Richards, C.M., Fozouni, P., et al. (2021). Genetic screens identify host factors for SARS-CoV-2 and common cold coronaviruses. Cell *184*, 106–119.e14. https://doi.org/10.1016/j.cell.2020.12.004.
5. Novick, P., Field, C., and Schekman, R. (1980). Identification of 23 complementation groups required for post-translational events in the yeast secretory pathway. Cell *21*, 205–215. https://doi.org/10.1016/0092-8674(80)90128-2.
6. TerBush, D.R., Maurice, T., Roth, D., and Novick, P. (1996). The exocyst is a multiprotein complex required for exocytosis in Saccharomyces cerevisiae. EMBO J. *15*, 6483–6494.
7. Hsu, S.C., Hazuka, C.D., Foletti, D.L., and Scheller, R.H. (1999). Targeting vesicles to specific sites on the plasma membrane: the role of the sec6/8 complex. Trends Cell Biol. *9*, 150–153.
8. Moskalenko, S., Tong, C., Rosse, C., Mirey, G., Formstecher, E., Daviet, L., Camonis, J., and White, M.A. (2003). Ral GTPases regulate exocyst assembly through dual subunit interactions. J. Biol. Chem. *278*, 51743–51748. https://doi.org/10.1074/jbc.M308702200.
9. Dubuke, M.L., Maniatis, S., Shaffer, S.A., and Munson, M. (2015). The exocyst subunit Sec6 interacts with assembled exocytic SNARE complexes. J. Biol. Chem. *290*, 28245–28256. https://doi.org/10.1074/jbc.M115.673806.
10. Yue, P., Zhang, Y., Mei, K., Wang, S., Lesigang, J., Zhu, Y., Dong, G., and Guo, W. (2017). Sec3 promotes the initial binary t-SNARE complex assembly and membrane fusion. Nat. Commun. *8*, 14236. https://doi.org/10.1038/ncomms14236.
11. Rosse, C., Formstecher, E., Boeckeler, K., Zhao, Y., Kremerskothen, J., White, M.D., Camonis, J.H., and Parker, P.J. (2009). An aPKC-exocyst complex controls paxillin phosphorylation and migration through localised JNK1 activation. PLoS Biol. *7*, e1000235. https://doi.org/10.1371/journal.pbio.1000235.
12. Rossi, G., Lepore, D., Kenner, L., Czuchra, A.B., Plooster, M., Frost, A., Munson, M., and Brennwald, P. (2020). Exocyst structural changes associated with activation of tethering downstream of Rho/Cdc42 GTPases. J. Cell Biol. *219*, https://doi.org/10.1083/jcb.201904161.
13. Ahmed, S.M., and Macara, I.G. (2017). The Par3 polarity protein is an exocyst receptor essential for mammary cell survival. Nat. Commun. *8*, 14867. https://doi.org/10.1038/ncomms14867.
14. Burke, J.E., Inglis, A.J., Perisic, O., Masson, G.R., McLaughlin, S.H., Rutaganira, F., Shokat, K.M., and Williams, R.L. (2014). Structures of PI4KIIIbeta complexes show simultaneous recruitment of Rab11 and its effectors. Science *344*, 1035–1038. https://doi.org/10.1126/science.1253397.
15. Aikawa, Y., and Martin, T.F. (2003). ARF6 regulates a plasma membrane pool of phosphatidylinositol(4,5)bisphosphate required for regulated exocytosis. J. Cell Biol. *162*, 647–659. https://doi.org/10.1083/jcb.200212142.
16. Honda, A., Nogami, M., Yokozeki, T., Yamazaki, M., Nakamura, H., Watanabe, H., Kawamoto, K., Nakayama, K., Morris, A.J., Frohman, M.A., and Kanaho, Y. (1999). Phosphatidylinositol 4-phosphate 5-kinase alpha is a downstream effector of the small G protein ARF6 in membrane ruffle formation. Cell *99*, 521–532. https://doi.org/10.1016/s0092-8674(00)81540-8.

17. Marquer, C., Tian, H., Yi, J., Bastien, J., Dall'Armi, C., Yang-Klingler, Y., Zhou, B., Chan, R.B., and Di Paolo, G. (2016). Arf6 controls retromer traffic and intracellular cholesterol distribution via a phosphoinositide-based mechanism. Nat. Commun. *7*, 11919. https://doi.org/10.1038/ncomms11919.

18. Shi, A., Liu, O., Koenig, S., Banerjee, R., Chen, C.C., Eimer, S., and Grant, B.D. (2012). RAB-10-GTPase-mediated regulation of endosomal phosphatidylinositol-4,5-bisphosphate. Proc. Natl. Acad. Sci. USA *109*, E2306–E2315. https://doi.org/10.1073/pnas.1205278109.

19. Prigent, M., Dubois, T., Raposo, G., Derrien, V., Tenza, D., Rossé, C., Camonis, J., and Chavrier, P. (2003). ARF6 controls post-endocytic recycling through its downstream exocyst complex effector. J. Cell Biol. *163*, 1111–1121. https://doi.org/10.1083/jcb.200305029.

20. Koumandou, V.L., Dacks, J.B., Coulson, R.M., and Field, M.C. (2007). Control systems for membrane fusion in the ancestral eukaryote; evolution of tethering complexes and SM proteins. BMC Evol. Biol. *7*, 29. https://doi.org/10.1186/1471-2148-7-29.

21. Yakir-Tamang, L., and Gerst, J.E. (2009). A phosphatidylinositol-transfer protein and phosphatidylinositol-4-phosphate 5-kinase control Cdc42 to regulate the actin cytoskeleton and secretory pathway in yeast. Mol. Biol. Cell *20*, 3583–3597. https://doi.org/10.1091/mbc.E08-10-1073.

22. Garrenton, L.S., Stefan, C.J., McMurray, M.A., Emr, S.D., and Thorner, J. (2010). Pheromone-induced anisotropy in yeast plasma membrane phosphatidylinositol-4,5-bisphosphate distribution is required for MAPK signaling. Proc. Natl. Acad. Sci. USA *107*, 11805–11810. https://doi.org/10.1073/pnas.1005817107.

23. Mizuno-Yamasaki, E., Medkova, M., Coleman, J., and Novick, P. (2010). Phosphatidylinositol 4-phosphate controls both membrane recruitment and a regulatory switch of the Rab GEF Sec2p. Dev. Cell *18*, 828–840. https://doi.org/10.1016/j.devcel.2010.03.016.

24. He, B., and Guo, W. (2009). The exocyst complex in polarized exocytosis. Curr. Opin. Cell Biol. *21*, 537–542. https://doi.org/10.1016/j.ceb.2009.04.007.

25. Thapa, N., Sun, Y., Schramp, M., Choi, S., Ling, K., and Anderson, R.A. (2012). Phosphoinositide signaling regulates the exocyst complex and polarized integrin trafficking in directionally migrating cells. Dev. Cell *22*, 116–130. https://doi.org/10.1016/j.devcel.2011.10.030.

26. Di Paolo, G., and De Camilli, P. (2006). Phosphoinositides in cell regulation and membrane dynamics. Nature *443*, 651–657. https://doi.org/10.1038/nature05185.

27. Wallroth, A., and Haucke, V. (2018). Phosphoinositide conversion in endocytosis and the endolysosomal system. J. Biol. Chem. *293*, 1526–1535. https://doi.org/10.1074/jbc.R117.000629.

28. Murray, D.H., and Tamm, L.K. (2009). Clustering of syntaxin-1A in model membranes is modulated by phosphatidylinositol 4,5-bisphosphate and cholesterol. Biochemistry *48*, 4617–4625. https://doi.org/10.1021/bi9003217.

29. An, S.J., Rivera-Molina, F., Anneken, A., Xi, Z., McNellis, B., Polejaev, V.I., and Toomre, D. (2021). An active tethering mechanism controls the fate of vesicles. Nat. Commun. *12*, 5434. https://doi.org/10.1038/s41467-021-25465-y.

30. Ketel, K., Krauss, M., Nicot, A.S., Puchkov, D., Wieffer, M., Müller, R., Subramanian, D., Schultz, C., Laporte, J., and Haucke, V. (2016). A phosphoinositide conversion mechanism for exit from endosomes. Nature *529*, 408–412. https://doi.org/10.1038/nature16516.

31. Campa, C.C., Margaria, J.P., Derle, A., Del Giudice, M., De Santis, M.C., Gozzelino, L., Copperi, F., Bosia, C., and Hirsch, E. (2018). Rab11 activity and PtdIns(3)P turnover removes recycling cargo from endosomes. Nat. Chem. Biol. *14*, 801–810. https://doi.org/10.1038/s41589-018-0086-4.

32. Farmer, T., Xie, S., Naslavsky, N., Stöckli, J., James, D.E., and Caplan, S. (2021). Defining the protein and lipid constituents of tubular recycling endosomes. J. Biol. Chem. *296*, 100190. https://doi.org/10.1074/jbc.RA120.015992.

33. Xiong, X., Xu, Q., Huang, Y., Singh, R.D., Anderson, R., Leof, E., Hu, J., and Ling, K. (2012). An association between type Igamma PI4P 5-kinase and Exo70 directs E-cadherin clustering and epithelial polarization. Mol. Biol. Cell *23*, 87–98. https://doi.org/10.1091/mbc.E11-05-0449.

34. Jouette, J., Guichet, A., and Claret, S.B. (2019). Dynein-mediated transport and membrane trafficking control PAR3 polarised distribution. eLife *8*, e40212. https://doi.org/10.7554/eLife.40212.

35. Liu, J., Zuo, X., Yue, P., and Guo, W. (2007). Phosphatidylinositol 4,5-bisphosphate mediates the targeting of the exocyst to the plasma membrane for exocytosis in mammalian cells. Mol. Biol. Cell *18*, 4483–4492. https://doi.org/10.1091/mbc.e07-05-0461.

36. He, B., Xi, F., Zhang, X., Zhang, J., and Guo, W. (2007). Exo70 interacts with phospholipids and mediates the targeting of the exocyst to the plasma membrane. EMBO J. *26*, 4053–4065. https://doi.org/10.1038/sj.emboj.7601834.

37. Zhang, X., Orlando, K., He, B., Xi, F., Zhang, J., Zajac, A., and Guo, W. (2008). Membrane association and functional regulation of Sec3 by phospholipids and Cdc42. J. Cell Biol. *180*, 145–158. https://doi.org/10.1083/jcb.200704128.

38. Mei, K., Li, Y., Wang, S., Shao, G., Wang, J., Ding, Y., Luo, G., Yue, P., Liu, J.J., Wang, X., et al. (2018). Cryo-EM structure of the exocyst complex. Nat. Struct. Mol. Biol. *25*, 139–146. https://doi.org/10.1038/s41594-017-0016-2.

39. Heider, M.R., Gu, M., Duffy, C.M., Mirza, A.M., Marcotte, L.L., Walls, A.C., Farrall, N., Hakhverdyan, Z., Field, M.C., Rout, M.P., et al. (2016). Subunit connectivity, assembly determinants and architecture of the yeast exocyst complex. Nat. Struct. Mol. Biol. *23*, 59–66. https://doi.org/10.1038/nsmb.3146.

40. Katoh, Y., Nozaki, S., Hartanto, D., Miyano, R., and Nakayama, K. (2015). Architectures of multisubunit complexes revealed by a visible immunoprecipitation assay using fluorescent fusion proteins. J. Cell Sci. *128*, 2351–2362. https://doi.org/10.1242/jcs.168740.

41. Lupas, A.N., and Bassler, J. (2017). Coiled Coils - A Model System for the 21st Century. Trends Biochem. Sci. *42*, 130–140. https://doi.org/10.1016/j.tibs.2016.10.007.

42. Ahmed, S.M., Nishida-Fukuda, H., Li, Y., McDonald, W.H., Gradinaru, C.C., and Macara, I.G. (2018). Exocyst dynamics during vesicle tethering and fusion. Nat. Commun. *9*, 5140. https://doi.org/10.1038/s41467-018-07467-5.

43. Bendezú, F.O., Vincenzetti, V., and Martin, S.G. (2012). Fission yeast Sec3 and Exo70 are transported on actin cables and localize the exocyst complex to cell poles. PLoS One *7*, e40248. https://doi.org/10.1371/journal.pone.0040248.

44. Lemaitre, R.P., Bogdanova, A., Borgonovo, B., Woodruff, J.B., and Drechsel, D.N. (2019). FlexiBAC: a versatile, open-source baculovirus vector system for protein expression, secretion, and proteolytic processing. BMC Biotechnol. *19*, 20. https://doi.org/10.1186/s12896-019-0512-z.

45. Ganesan, S.J., Feyder, M.J., Chemmama, I.E., Fang, F., Rout, M.P., Chait, B.T., Shi, Y., Munson, M., and Sali, A. (2020). Integrative structure and function of the yeast exocyst complex. Protein Sci. *29*, 1486–1501. https://doi.org/10.1002/pro.3863.

46. Picco, A., Irastorza-Azcarate, I., Specht, T., Böke, D., Pazos, I., Rivier-Cordey, A.S., Devos, D.P., Kaksonen, M., and Gallego, O. (2017). The in vivo architecture of the exocyst provides structural basis for exocytosis. Cell *168*, 400–412.e18. https://doi.org/10.1016/j.cell.2017.01.004.

47. Zhai, Y., Zhang, D., Yu, L., Sun, F., and Sun, F. (2019). SmartBac, a new baculovirus system for large protein complex production. J. Struct. Biol. X *1*, 100003. https://doi.org/10.1016/j.yjsbx.2019.100003.

48. Desclozeaux, M., Venturato, J., Wylie, F.G., Kay, J.G., Joseph, S.R., Le, H.T., and Stow, J.L. (2008). Active Rab11 and functional recycling endosome are required for E-cadherin trafficking and lumen formation during epithelial morphogenesis. Am. J. Physiol. Cell Physiol. *295*, C545–C556. https://doi.org/10.1152/ajpcell.00097.2008.

49. Simons, K., and Wandinger-Ness, A. (1990). Polarized sorting in epithelia. Cell *62*, 207–210. https://doi.org/10.1016/0092-8674(90)90357-k.

50. Murray, D.H., Jahnel, M., Lauer, J., Avellaneda, M.J., Brouilly, N., Cezanne, A., Morales-Navarrete, H., Perini, E.D., Ferguson, C., Lupas, A.N., et al. (2016). An endosomal tether undergoes an entropic collapse to bring vesicles together. Nature *537*, 107–111. https://doi.org/10.1038/nature19326.

51. Pucadyil, T.J., and Schmid, S.L. (2010). Supported bilayers with excess membrane reservoir: a template for reconstituting membrane budding and fission. Biophys. J. *99*, 517–525. https://doi.org/10.1016/j.bpj.2010.04.036.

52. Hansen, S.D., Huang, W.Y.C., Lee, Y.K., Bieling, P., Christensen, S.M., and Groves, J.T. (2019). Stochastic geometry sensing and polarization in a lipid kinase-phosphatase competitive reaction. Proc. Natl. Acad. Sci. USA *116*, 15013–15022. https://doi.org/10.1073/pnas.1901744116.

53. Kolossov, V.L., Sivaguru, M., Huff, J., Luby, K., Kanakaraju, K., and Gaskins, H.R. (2018). Airyscan super-resolution microscopy of mitochondrial morphology and dynamics in living tumor cells. Microsc. Res. Tech. *81*, 115–128. https://doi.org/10.1002/jemt.22968.

54. Hammond, G.R., and Balla, T. (2015). Polyphosphoinositide binding domains: key to inositol lipid biology. Biochim. Biophys. Acta *1851*, 746–758. https://doi.org/10.1016/j.bbalip.2015.02.013.

55. Klima, M., Tóth, D.J., Hexnerova, R., Baumlova, A., Chalupska, D., Tykvart, J., Rezabkova, L., SenGupta, N., Man, P., Dubankova, A., et al. (2016). Structural insights and in vitro reconstitution of membrane targeting and activation of human PI4KB by the ACBD3 protein. Sci. Rep. *6*, 23641. https://doi.org/10.1038/srep23641.

56. Lemmon, M.A., Ferguson, K.M., O'Brien, R., Sigler, P.B., and Schlessinger, J. (1995). Specific and high-affinity binding of inositol phosphates to an isolated pleckstrin homology domain. Proc. Natl. Acad. Sci. USA *92*, 10472–10476. https://doi.org/10.1073/pnas.92.23.10472.

57. D'Souza-Schorey, C., Li, G., Colombo, M.I., and Stahl, P.D. (1995). A regulatory role for ARF6 in receptor-mediated endocytosis. Science *267*, 1175–1178. https://doi.org/10.1126/science.7855600.

58. Radhakrishna, H., Al-Awar, O., Khachikian, Z., and Donaldson, J.G. (1999). ARF6 requirement for Rac ruffling suggests a role for membrane trafficking in cortical actin rearrangements. J. Cell Sci. *112*, 855–866.

59. Brown, F.D., Rozelle, A.L., Yin, H.L., Balla, T., and Donaldson, J.G. (2001). Phosphatidylinositol 4,5-bisphosphate and Arf6-regulated membrane traffic. J. Cell Biol. *154*, 1007–1017. https://doi.org/10.1083/jcb.200103107.

60. Krauss, M., Kinuta, M., Wenk, M.R., De Camilli, P., Takei, K., and Haucke, V. (2003). ARF6 stimulates clathrin/AP-2 recruitment to synaptic membranes by activating phosphatidylinositol phosphate kinase type Igamma. J. Cell Biol. *162*, 113–124. https://doi.org/10.1083/jcb.200301006.

61. Beutel, O., Nikolaus, J., Birkholz, O., You, C., Schmidt, T., Herrmann, A., and Piehler, J. (2014). High-fidelity protein targeting into membrane lipid microdomains in living cells. Angew. Chem. Int. Ed. Engl. *53*, 1311–1315. https://doi.org/10.1002/anie.201306328.

62. Shinar, G., Dekel, E., Tlusty, T., and Alon, U. (2006). Rules for biological regulation based on error minimization. Proc. Natl. Acad. Sci. USA *103*, 3999–4004. https://doi.org/10.1073/pnas.0506610103.

63. Brandman, O., and Meyer, T. (2008). Feedback loops shape cellular signals in space and time. Science *322*, 390–395. https://doi.org/10.1126/science.1160617.

64. Lee, C., Lepore, D., Munson, M., and Yoon, T.-Y. (2022). Exocyst stimulates each step of exocytic SNARE complex assembly and vesicle fusion. bioRxiv. 2022.2001.2016.476540. https://doi.org/10.1101/2022.01.16.476540.

65. Tan, X., Thapa, N., Choi, S., and Anderson, R.A. (2015). Emerging roles of PtdIns(4,5)P2–beyond the plasma membrane. J. Cell Sci. *128*, 4047–4056. https://doi.org/10.1242/jcs.175208.

66. Marchesin, V., Castro-Castro, A., Lodillinsky, C., Castagnino, A., Cyrta, J., Bonsang-Kitzis, H., Fuhrmann, L., Irondelle, M., Infante, E., Montagnac, G., et al. (2015). ARF6-JIP3/4 regulate endosomal tubules for MT1-MMP exocytosis in cancer invasion. J. Cell Biol. *211*, 339–358. https://doi.org/10.1083/jcb.201506002.

67. Santana-Molina, C., Gutierrez, F., and Devos, D.P. (2021). Homology and modular evolution of CATCHR at the origin of the eukaryotic endomembrane system. Genome Biol. Evol. *13*, https://doi.org/10.1093/gbe/evab125.

68. Bodemann, B.O., Orvedahl, A., Cheng, T., Ram, R.R., Ou, Y.H., Formstecher, E., Maiti, M., Hazelett, C.C., Wauson, E.M., Balakireva, M., et al. (2011). RalB and the exocyst mediate the cellular starvation response by direct activation of autophagosome assembly. Cell *144*, 253–267. https://doi.org/10.1016/j.cell.2010.12.018.

69. Singh, S., Kumari, R., Chinchwadkar, S., Aher, A., Matheshwaran, S., and Manjithaya, R. (2019). Exocyst subcomplex functions in autophagosome biogenesis by regulating Atg9 trafficking. J. Mol. Biol. *431*, 2821–2834. https://doi.org/10.1016/j.jmb.2019.04.048.

70. Zaman, A., Wu, X., Lemoff, A., Yadavalli, S., Lee, J., Wang, C., Cooper, J., McMillan, E.A., Yeaman, C., Mirzaei, H., et al. (2021). Exocyst protein subnetworks integrate Hippo and mTOR signaling to promote virus detection and cancer. Cell Rep. *36*, 109491. https://doi.org/10.1016/j.celrep.2021.109491.

71. Mao, L., Zhan, Y.Y., Wu, B., Yu, Q., Xu, L., Hong, X., Zhong, L., Mi, P., Xiao, L., Wang, X., et al. (2020). ULK1 phosphorylates Exo70 to suppress breast cancer metastasis. Nat. Commun. *11*, 117. https://doi.org/10.1038/s41467-019-13923-7.

72. Uhm, M., Bazuine, M., Zhao, P., Chiang, S.H., Xiong, T., Karunanithi, S., Chang, L., and Saltiel, A.R. (2017). Phosphorylation of the exocyst protein Exo84 by TBK1 promotes insulin-stimulated GLUT4 trafficking. Sci. Signal. *10*, https://doi.org/10.1126/scisignal.aah5085.

73. Liu, D., Li, X., Shen, D., and Novick, P. (2018). Two subunits of the exocyst, Sec3p and Exo70p, can function exclusively on the plasma membrane. Mol. Biol. Cell *29*, 736–750. https://doi.org/10.1091/mbc.E17-08-0518.

74. Hartman, E.J., Asady, B., Romano, J.D., and Coppens, I. (2022). The Rab11-Family Interacting proteins reveal selective interaction of mammalian recycling endosomes with the *Toxoplasma* parasitophorous vacuole in a Rab11- and Arf6-dependent manner. Mol. Biol. Cell *5*, ar34. https://doi.org/10.1091/mbc.E21-06-0284.

75. Kobayashi, H., and Fukuda, M. (2013). Arf6, Rab11 and transferrin receptor define distinct populations of recycling endosomes. Commun. Integr. Biol. *6*, e25036. https://doi.org/10.4161/cib.25036.

76. Shiba, T., Koga, H., Shin, H.W., Kawasaki, M., Kato, R., Nakayama, K., and Wakatsuki, S. (2006). Structural basis for Rab11-dependent membrane recruitment of a family of Rab11-interacting protein 3 (FIP3)/Arfophilin-1. Proc. Natl. Acad. Sci. USA *103*, 15416–15421. https://doi.org/10.1073/pnas.0605357103.

77. Schneider, C.A., Rasband, W.S., and Eliceiri, K.W. (2012). NIH Image to ImageJ: 25 years of image analysis. Nat. Methods *9*, 671–675. https://doi.org/10.1038/nmeth.2089.

78. Kremer, J.R., Mastronarde, D.N., and McIntosh, J.R. (1996). Computer visualization of three-dimensional image data using IMOD. J. Struct. Biol. *116*, 71–76. https://doi.org/10.1006/jsbi.1996.0013.

79. Tang, G., Peng, L., Baldwin, P.R., Mann, D.S., Jiang, W., Rees, I., and Ludtke, S.J. (2007). EMAN2: an extensible image processing suite for electron microscopy. J. Struct. Biol. *157*, 38–46. https://doi.org/10.1016/j.jsb.2006.05.009.

80. Rizk, A., Paul, G., Incardona, P., Bugarski, M., Mansouri, M., Niemann, A., Ziegler, U., Berger, P., and Sbalzarini, I.F. (2014). Segmentation and quantification of subcellular structures in fluorescence microscopy images using Squassh. Nat. Protoc. *9*, 586–596. https://doi.org/10.1038/nprot.2014.037.

CellPress
OPEN ACCESS

81. Mastronarde, D.N. (2005). Automated electron microscope tomography using robust prediction of specimen movements. J. Struct. Biol. *152*, 36–51. https://doi.org/10.1016/j.jsb.2005.07.007.

82. Hagen, W.J.H., Wan, W., and Briggs, J.A.G. (2017). Implementation of a cryo-electron tomography tilt-scheme optimized for high resolution subtomogram averaging. J. Struct. Biol. *197*, 191–198. https://doi.org/10.1016/j.jsb.2016.06.007.

83. Lord, S.J., Velle, K.B., Mullins, R.D., and Fritz-Laylin, L.K. (2020). SuperPlots: communicating reproducibility and variability in cell biology. J. Cell Biol. *219*, https://doi.org/10.1083/jcb.202001064.

STAR★METHODS

KEY RESOURCES TABLE

REAGENT or RESOURCE	SOURCE	IDENTIFIER
Antibodies		
Mouse anti E-Cadherin Clone 36	BD Bioscience	Cat#610181; RRID:AB_397580
Purified Mouse Anti-GM130	BD Bioscience	Cat#610822; RRID:AB_398141
Bacterial and virus strains		
E. Coli DH5α	SLS-reagents	N/A
E. Coli BL21	SLS-reagents	N/A
Chemicals, peptides, and recombinant proteins		
Glutathione Agarose	SLS-reagents	N/A
Amylose resin	SLS-reagents	N/A
Quick Coomassie Stain	Generon	Cat#NB-45-00078
NuPAGE™ 4 to 12% gradient Protein Gel	ThermoFisher	Cat#NP0321BOX
POPC (16:0/18:1 PC)	Avanti	Cat#850457
POPS (16:0/18:1 PS)	Avanti	Cat#840034
PI(4)P diC16	Echelon	Cat#P-4016
PI(4,5)P_2 diC16	Echelon	Cat#P-4516
Atto 647N DOPE	Sigma-Aldrich	Cat#42247
Rhodamine DPPE	Avanti	Cat#810150C
NBD DPPE	Avanti	Cat#810144P
Tris-NTA-DODA	Beutel et al.[61]	N/A
Monodispersed silica standards 10μm beads	Whitehouse Scientific	Cat#MSS010
Insect-XPRESS	Lonza	Cat#BELN12-730Q
ESCORT IV	Sigma-Aldrich	Cat#L3287
HEPES	SLS-reagents	N/A
NaCl	SLS-reagents	N/A
TCEP	Generon	Cat#GEN-TCEP-10
Na_2HPO_4	Sigma-Aldrich	Cat#S9763
Benzonase	Sigma-Aldrich	Cat#E1014
Lactose	Fisher	Cat#10375850
Uranyl formate	Electron Microscopy Supplies	Cat#22450
Nickel chloride	Sigma-Aldrich	Cat#339350
$MgCl_2$	SLS-reagents	N/A
EGTA	SLS-reagents	N/A
Casein	Sigma-Aldrich	Cat#C6905
BME	Sigma-Aldrich	Cat#M6250
Glucose	Sigma-Aldrich	Cat#D9434
ATP	Sigma-Aldrich	Cat#A1852
DMEM	ThermoFisher	Cat#41966052
FBS	ThermoFisher	Cat#10270106
PenStrep	ThermoFisher	Cat#15140-122
NMuMG-optimized GeneJet	SignaGen Laboratories	Cat#SL100489-NMuMG
PBS	ThermoFisher	Cat#14190-169
PFA	ThermoFisher	Cat#28908
NH4Cl	Sigma-Aldrich	Cat#A9434
Digitonin	Sigma-Aldrich	Cat#D141
PIPES	Sigma-Aldrich	Cat#P8203

(Continued on next page)

Continued

REAGENT or RESOURCE	SOURCE	IDENTIFIER
ProLong Gold	Fisher	Cat#P36934
Goat serum	Sigma-Aldrich	Cat#G9023
Saponin	Sigma-Aldrich	Cat#47036
Glutathione	Sigma-Aldrich	Cat#G4251
Critical commercial assays		
Pierce™ Coomassie (Bradford) Protein Assay Kit	ThermoFisher	Cat#23200
Experimental models: Cell lines		
NMuMG cells	ATCC	Cat#CRL-1636
NMuMG Exoc4-sfGFP Knock In	Ahmed et al.[42]	N/A
Recombinant DNA		
DefBac viral backbone	Lemaitre et al.[44]	N/A
pOCC82 no tag	Lemaitre et al.[44]	N/A
pOCC82-Exoc1	This paper	N/A
pOCC82-Exoc2	This paper	N/A
pOCC82-Exoc3	This paper	N/A
pOCC82-Exoc4	This paper	N/A
pOCC82-Exoc5	This paper	N/A
pOCC82-Exoc6	This paper	N/A
pOCC82-Exoc7	This paper	N/A
pOCC82-Exoc8	This paper	N/A
pOCC151 N terminal GST	Lemaitre et al.[44]	N/A
pOCC151-Exoc1	This paper	N/A
pOCC151-Exoc2	This paper	N/A
pOCC151-Exoc3	This paper	N/A
pOCC151-Exoc4	This paper	N/A
pOCC151-Exoc5	This paper	N/A
pOCC151-Exoc6	This paper	N/A
pOCC151-Exoc7	This paper	N/A
pOCC151-Exoc8	This paper	N/A
pOCC102 N terminal MBP	Lemaitre et al.[44]	N/A
pOCC102-Exoc4	This paper	N/A
pOCC102-Exoc5	This paper	N/A
pOCC102-PIP5K1C	This paper	N/A
pOCC285 N terminal mNeonGreen	Lemaitre et al.[44]	N/A
pOCC285-Exoc4	This paper	N/A
pOCC285-Exoc6	This paper	N/A
pcDNA3 PIP5K1C-mScarlet	This paper	N/A
pcDNA3 Arf6-Q67L mRuby3	This paper	N/A
pcDNA3 Arf6-T27N mRuby3	This paper	N/A
pcDNA3 Arf6-WT mRuby3	This paper	N/A
pGEX-6P-1 Arf6-Q67L (N-terminal GST)	This paper	N/A
pGEX-6P-1 Arf6-T27N (N-terminal GST)	This paper	N/A
pOCC3 Arf6-Q67L (N-terminal 6xHis)	This paper	N/A
pOCC3 Arf6-T27N (N-terminal 6xHis)	This paper	N/A
pOCC10-SidC (N-terminal eGFP)	This paper	N/A
pOCC10-PLCδ-PH (N-terminal eGFP)	This paper	N/A
pOCC15-PLCδ-PH (N-terminal tagRFP)	This paper	N/A
GFP-PIPK1 gamma 90	Addgene	Cat#22299

(*Continued on next page*)

Continued		
REAGENT or RESOURCE	SOURCE	IDENTIFIER
Software and algorithms		
ImageJ	Schneider et al.[77]	https://imagej.nih.gov/ij/
Prism 9	Graphpad	https://www.graphpad.com
IMOD, 3dmod	Kremer et al.[78]	https://bio3d.colorado.edu/imod/
EMAN2	Tang et al.[79]	https://blake.bcm.edu/emanwiki/EMAN2
Squassh	Rizk et al.[80]	https://imagej.net/plugins/squassh

RESOURCE AVAILABILITY

Lead contact

Please contact the lead contact, Dr. David H Murray, for additional information and requests for resources and reagents (dhmurray@dundee.ac.uk).

Materials availability

All plasmids generated in this study are available from the lead contact with a completed Materials Transfer Agreement

Data and code availability

- Original full Coomassie gels are available in the Data S1 file. Microscopy data reported in this paper will be shared by the lead contact upon request
- No code was generated in this study.
- Any additional information required to reanalyze the data reported in this paper is available from the lead contact upon request

EXPERIMENTAL MODEL AND SUBJECT DETAILS

NMuMG cell line are derived from female, *Mus musculus* mammary gland and were acquired from ATCC. Cells were grown in Dulbecco's modified Eagle's medium with 4.5 g/L glucose and 10% fetal bovine serum with addition of Pen/Strep. NMuMG cells with endogenous Exoc4-sfGFP knock-in were a kind gift of Ian Macara and previously described in Ahmed et al.[42]. Cells were grown at 37C in 5% CO_2 incubators and passaged every 2nd day in a 1/5 ration.

Sf9 insect cells derived from *Spodoptera frugiperda* were acquired from ThermoFisher, grown in ESF921 insect cell media at 27C in suspension at constant orbital shaking and split every 2nd day at a cell count at 1x10^6 cells/ml.

All *in vitro* reconstituted material was designed and purified based on human sequences.

METHOD DETAILS

Plasmid construction

Exocyst subunit plasmids were a kind gift from Channing Der (Addgene #53755-53762). Each was subcloned into FlexiBAC SF9 expression plasmids as GST-HRV3C, 6xHis-MBP-HRV3C, and 6xHis-HRV3C-mNeonGreen fusions, where HRV3C is a protease cleavage site, by standard molecular biology approaches.[44] GFP-PIPK1 gamma 90 was a gift from Pietro De Camilli (Addgene #22299) and subcloned with mScarlet-I into mammalian expression plasmids. Plasmids encoding PLCδ-PH and SidC were a kind gift from Evzen Boura and subcloned into bacterial expression plasmids. Gene encoding Arf6 was cloned into bacterial expression plasmids as a GST-fusion, and subcloned with mRuby3 into mammalian expression plasmids.

Baculovirus production

Exocyst subunits and PIP5K1C were expressed in SF9 cells cultured in Insect-XPRESS (Lonza) by baculovirus mediated expression. Baculovirus was produced using the FlexiBac methods.[44] SF9 cells at 1x10^6 cells/ml were co-transfected by the linearised Defbac viral backbone and the plasmids encoding for exocyst subunits or PIP5K1C using ESCORT-IV (Sigma). After 5 days, cells were checked for signs of viral transfection and 50-200 μl of supernatant of this P1 virus were used to infect 50 ml of insect cells to generate P2 virus. 5 days after infection, this P2 virus was again evaluated for virus production (LUNA-II; Logos Bio).

Exocyst subunit interaction screen

For identification of exocyst subunit interactions, SF9 cells at 1x10^6 SF9 cells/ml were grown in 24 well round bottom plates (Qiagen) and infected with baculovirus of the indicated single subunits. After 2 days of infection, the cells were pelleted at 500xg for 10 min, the supernatant discarded and the pellet frozen in liquid nitrogen. Upon use, the pellet was thawed and resuspended in 2 ml of standard buffer (20mM HEPES, 250mM NaCl, 0.5 mM TCEP), sonicated, and clarified at 4 °C. 25 μl of glutathione agarose beads (MRC-PPU

Reagents and Services) were added and incubated for 1-2 h at 4 °C. Beads were isolated by centrifugation and washed three times in standard buffer using MobiSpin Column F (MoBiTec GmbH). The washed beads were resuspended in 50 μl 1x SDS loading buffer, boiled at 95 °C, and separated by SDS-PAGE. Gels were stained with Quick Coomassie Stain (Generon) and scanned.

Protein purifications

Exocyst complexes and PIP5K1C were expressed using the SF9 insect cell system.[44] To express exocyst Subcomplex-1, cells were coinfected with N-terminal 6xHis-MBP tagged Exoc1 and untagged Exoc2. Separately, cells were coinfected with untagged Exoc3 and N-terminal 6xHis-mNeonGreen tagged Exoc4. Exoc1 was infected at 0.9 relative MOI relative to the other subunits to assure complex integration, with each virus having similar MOI. After two days of infection, the cultures were mixed, pelleted and frozen in liquid nitrogen.

To express exocyst Subcomplex-2, cells were coinfected with N-terminal 6xHis-mNeonGreen tagged Exoc6 and N-terminal 6xHis-MBP tagged Exoc5 at 0.8 relative MOI. Separately, untagged Exoc7 and untagged Exoc8 were coexpressed at 1.2 relative MOI. After two days of infection, the cultures were mixed, pelleted and frozen in liquid nitrogen.

To express exocyst holocomplex, cells were coinfected with untagged Exoc1 and untagged Exoc2, and separately with untagged Exoc3 and untagged Exoc4. Separately, cells were coinfected with N-terminal 6xHis-MBP tagged Exoc5 at 0.8 relative MOI, and N-terminal 6xHis-mNeonGreen tagged Exoc6. Finally, a final separate culture was infected with untagged Exoc7 and untagged Exoc8 at 1.2 relative MOI. After two days of infection, each of the 4 cultures was combined, pelleted and frozen in liquid nitrogen. In our hands, the MOI ratio of tagged subunit was of critical importance for generation of stoichiometric complexes.

To express PIP5K1C, cells were infected with N-terminal 6xHis-MBP tagged PIP5K1C for two days, pelleted and frozen in liquid nitrogen.

To purify the exocyst complexes, all pellets were thawed on ice and resuspended to 50ml final volume in standard buffer with the addition of protease inhibitors and Benzonase (Sigma). PIP5K1C was resuspended in buffer containing 20 mM HEPES, 400 mM NaCl, 0.5 mM TCEP, 50 mM Na_2HPO_4. Cell suspensions were lysed by Dounce homogenization followed by clarification through centrifugation. Cleared lysate was filtered through 0.45 μm membranes and bound to preequilibrated Amylose Fast Flow resin (MRC-PPU Reagents and Services). After washing, protein/proteins complexes were cleaved by addition of recombinant HRV-3C protease for at least 3 hours. Following cleavage and centrifugation, the supernatant was concentrated to using Vivaspin concentrators (Cytiva) and injected onto a 24 ml Superose 6 Increase 10/300 GL (or Superdex200 10/300 GL for Exoc1:mNeonGreen-Exoc4 and Exoc5:mNeonGreen-Exoc6:Exoc7) equilibrated in standard buffer. Peak fractions were analyzed by SDS-PAGE, pooled and concentrated and either used immediately in experiments, or aliquoted and frozen in liquid nitrogen and stored at -80 °C. Samples were sent to the mass spectrometry facility (Fingerprints, University of Dundee) for quantitative peptide analysis.

Small GTPases and lipid probes were expressed in BL21 bacterial cells using standard approaches. All constructs were grown in LB containing 1.75 w/vol % lactose and antibiotic at 37 °C to OD_{600}=0.8, whereupon temperature was lowered to 18 °C for 10-12 h. Cells were pelleted resuspended in standard buffer, lysed, and clarified. Protein was purified by Ni affinity chromatography using 5 ml His-Trap HP column (Cytiva) followed by anion exchange on a 5ml Capto-Q column (Cytiva). Peak fractions were pooled and purified by size exclusion chromatography using a Superdex 200 16/60 pg column (Cytiva). All proteins were aliquoted, frozen in liquid nitrogen and stored at -80 °C.

Protein concentrations for purified exocyst complexes and PIP5K1C were determined using a Pierce™ Coomassie (Bradford) Protein Assay Kit across several dilutions using systematically and independently generated standard curves each time. Protein concentrations for purified Arf6 were determined by measuring absorbance at 280 nm using a Nanodrop (Thermofisher) and calculated extinction coefficients.

Negative stain electron microscopy

Carbon Film, 400 Mesh copper grids (Electron Microscopy Sciences) were glow discharged and exocyst holocomplex (1.9 μM) was applied for 10 minutes and blotted away with Whatman paper. Grids where washed twice with ddH_2O before being stained with 0.75% uranyl formate for approximately 30s. Excess stain was removed by blotting, and samples were imaged using a JEOL 2200FS 200 kV with inline omega filter on a Gatan Ultrascan 4k 15 μm pixel camera at 30,000x magnification. Images were obtained at a defocus of -0.5-2.0 μm. Particles were manually selected using Boxer implemented in EMAN2.[79] Morphological model was generated in EMAN2 from 7052 particles over 75 micrographs to assess protein complex heterogeneity and dimensions.

Lipids, membrane-coated beads, and lipid binding experiments

All lipids were obtained from Avanti Polar Lipids unless otherwise stated. Liposomes containing either PI(4)P (Echelon Biosciences) or $PI(4,5)P_2$ (Echelon Biosciences) were produced by mixing 85 mol % 1-palmitoyl-2-oleoyl-glycero-3-phosphocholine (POPC), 10 mol % phosphatidylserine (POPS) with 5 mol % of the respective phosphatidylinositol together with 0.1% Atto647N-DOPE (ATTO-TEC) or Rhodamine-DOPE. Control liposomes without phosphoinositides contain 90 mol% POPC, 10 mol% POPS and 0.1% Atto647N-DOPE (ATTO-TEC) or Rhodamine-DOPE. The mixtures were evaporated under nitrogen flow and dried overnight in a vacuum. Dried lipids were resuspended in buffer containing 20 mM HEPES, 150 mM NaCl, and 0.5 mM TCEP at 37 °C and subjected to 6 cycles of freeze thawing in liquid nitrogen. Where samples contained 0.5% Tris-NTA-DODA[61] for Arf6 conjugation, 1 mM $NiCl_2$ was added during the resuspension step. Liposomes were extruded to 100 nm and aliquoted, frozen in liquid nitrogen and stored at -20 °C.

Membrane-coated beads[50,51] were generated by mixing liposomes and 10 μm silica beads (Whitehouse Scientific) in 200 mM NaCl. Beads were washed twice with 20 mM HEPES and resuspended in buffer containing 20 mM HEPES, 150 mM NaCl, and 0.5mM TCEP. Samples were kept on a rotator until use within 2 hours of generation.

To assess lipid binding, beads were added to uncoated μ-Slide 8 well chambers (Ibidi) and protein added and mixed by pipetting at the indicated final concentrations. Lipid binding kinetics were allowed to equilibrate for 30 min at room temperature before imaging at the transverse plane.

Confocal images were acquired using a Leica SP8 Confocal Microscope with a Leica HC PL APO CS2 63x/1.40 Oil objective at 0.75 base zoom with 1024x1024 pixels scan.

Phosphoinositide conversion assays

Membrane-coated beads containing 0.1% Atto647N-DOPE (ATTO-TEC), 5% PI(4)P and 10% POPS in 84.9% POPC were prepared as described above and resuspended in 100 μl kinase buffer (20 mM HEPES (pH 7.0), 150 mM NaCl, 5 mM $MgCl_2$, 0.5 mM EGTA, 200 μg/mL β-casein, 20 mM BME, and 20 mM glucose) in the presence or absence of 1 mM ATP. Samples were applied to an imaging chamber and protein was added to a final concentration of 100 nM. Immediately before acquisition, reconstituted PIP5K1C was added to the beads at a final concentration of 150 nM. The reaction was monitored at 0.2 Hz using a Leica SP8 Confocal Microscope with a Leica HC PL APO CS2 63x/1.40 Oil objective at 0.75 base zoom with 1024x1024 pixel scan.

For membrane tethering assays, liposomes containing 5% $PI(4,5)P_2$ were added to the beads together with PIP5K1C for 30 min while rotation at RT before imaging.

For Arf6 mediated phosphoinositide conversion, 1 μM purified 6xHis-Arf6-T27N/Q67L was added to membrane-coated beads containing 0.5% Tris-NTA-DODA,[61] 5% PI(4)P, 10% POPS in 84.5% POPC doped with either 0.1% NBD-DPPE or Atto647N-DOPE (ATTO-TEC) for 30 min and washed three times. At these conditions Arf6 binding to the beads were saturated. Beads were added to an Ibidi imaging slide and purified PLCδ-PH domain with N-terminal RFP tag were added to a final concentration of 100 nM. Immediately before imaging, purified PIP5K1C was added to the beads at a final concentration of 12.5 nM. Recruitment of PLCδ to either of the two membrane-coated bead populations was determined using ImageJ[77] by segmentation of either the NBD or Atto647N signal as mask.

Cryo-electron tomography

Exocyst holocomplex was mixed with liposomes composed of 95 mol% POPC and 5 mol% $PI(4,5)P_2$ and protein A coated 5 nm gold fiducials (Electron Microscopy Sciences) for 3 minutes and to a final protein concentration of 1 μM. Samples were applied to glow-discharged holey carbon film grids (Quantifoil) and plunge frozen into liquid ethane using a Vitrobot (ThermoFisher). Tilt series were collected on a JEOL CRYO ARM 300 (Jeol Ltd) electron microscope operated at 300 keV and equipped with in-column omega energy filter and DE-64 direct electron detector (Direct Electron, USA) operated in electron counting mode. Data acquisition was controlled by SerialEM[81] using the dose-symmetric scheme[82] using an increment of 1° and a group size of 3° with a range of -60° to +60°. The nominal magnification was 30,000x, giving a calibrated pixel size of 2.35 Å/px. The defocus was set to -6 μm and the dose was 1.84 e/Å2/tilt giving a total dose of 223 e/Å2 over a complete tilt series. The energy filter was set to a slit width of 30 eV. Images were collected as movies of 6 frames per tilt. Movies were motion corrected, gain corrected, assembled into tilt series and dose weighted using the IMOD program alignframes.[81] Tomograms were reconstructed by back projection using the IMOD program ETOMO with 5 nm gold particles as fiducials. Liposomes were segmented by hand throughout the tilt series in 3dmod[78] and the distance of closest approach was determined. Proteinaceous material that was clearly associated with liposomes was segmented using the AutoThreshold function.

Live-cell Airyscan imaging

NMuMG cells (ATCC) and NMuMG genome-modified cells[42] were grown in DMEM containing 4.5 μg/ml glucose with pyruvate, supplemented with 10% FBS and PenStrep. Cells were seeded onto Ibidi glass bottom dishes and transfected using NMuMG-optimized GeneJet transfection reagent (SignaGen) according to the manufacturer's instructions. 24-48 h after transfection cells were imaged in an environmental control chamber at 37 C with 5% CO_2 using a Zeiss LSM880 Airyscan microscope with a 60x oil objective NA1.4.

Phosphoinositide staining in cells

Staining protocols for phosphoinositides using purified SidC-GFP and GFP-PLCδ-PH were adopted from Hammond et al. 2019. For staining with purified SidC-GFP, cells were fixed with 37 °C prewarmed PBS with 4% PFA for 20 min, followed by quenching in 50 mM NH4Cl for 20 min. Fixed cells were permeabilised with 20 μM digitonin in 1X PIPES buffer for 5 min at room temperature and washed. Fixed and permeabilised cells were blocked with 5% goat serum in 50 mM NH4Cl containing PIPES buffer in the presence of 1 μM purified SidC-GFP for 1 h at room temperature. Cells were washed in PIPES buffer and stained with mouse anti-GM130 (BD Biosciences 610822) for 1 h, washed and stained with secondary antibodies coupled to Alexa 647 for 45 min, washed and postfix in PBS with 2% PFA for 10 min. Samples were washed in 50 mM NH4Cl, mounted using Prolong Gold (ThermoFisher) and imaged using an LSM880 Airyscan microscope with a 60x, NA1.4 oil objective.

For staining with purified GFP-PLCδ-PH, cells were fixed with prewarmed 37C 4% PFA in PBS for 20min, followed by quenching in 50 mM NH4Cl for 20 min. From now on all steps were carried out on ice and with all solutions chilled to 4 °C. Fixed cells were permeabilised with 0.5% saponin in PIPES buffer with 5% goat serum and 2 μM purified GFP-PLCδ-PH for 1 h. Fixed and permeabilised

cells were washed with PIPES buffer and stained with mouse anti-E-cadherin (Clone 36/E; BD Bioscience) in 5% goat serum and 0.1% saponin containing PIPES buffer for 1 h washed and stained with secondary antibodies coupled to Alexa 647 for 45 min. Ice cold PBS with 2% PFA was added for 10 min before transferring the slides to room temperature and letting them adjust for another 10 min. Samples were rinsed in 50 mM NH4Cl, mounted, and imaged as above described.

Arf6 - PIP5K pulldown

BL21 cells transformed with GST alone or GST-Arf6-Q67L/T27N were grown in LB containing 1.75% lactose and antibiotic at 37 °C to OD_{600}=0.8, whereupon temperature was lowered to 18 °C for 10-12 h. Cells were pelleted, resuspended in standard buffer, lysed, and clarified. Sample was then filtered through a 0.22 μm filter and bound to GSH agarose beads (MRC-PPU Reagents and Services) for 1 h at 4 °C. Beads were washed, and protein was eluted with 20 μM glutathione. Eluted protein was further purified by size exclusion chromatography using a Superdex 200 16/60 pg column. Peak fractions were pooled and snap frozen in liquid nitrogen. Equal amounts of purified protein were added to 50 μl GSH agarose for 1 h at 4 °C, washed and resuspended in standard buffer. Purified PIP5K1C was added to a final concentration of 400 nM and incubated at 4 °C under constant rotation for 1 h. Beads were washed, resuspended in SDS-loading buffer and analysed by SDS page, followed by Coomassie staining.

QUANTIFICATION AND STATISTICAL ANALYSIS

Membrane coated bead assays were analysed using a custom written ImageJ script. The signal from the Atto647N-DOPE or Rhodamine-DOPE was used to segment each bead individually, thereby creating a mask for the circumference for bead. The mask was used in the GFP/mNeonGreen channel to measure the recruitment of the fluorescent probes to each bead. Statistical details, including n numbers and repeats can be found in the corresponding figure legends. Statistics for determination of relative binding constants were acquired in GraphPad9 as single-site model, probability by Aoike Information Criterion <0.01%.

The amount of signal overlap from the cell-based imaging approaches was determined using ImageJ. Overlap between the different channels was quantified using the automated segmentation plugin Squassh[80] and plotted as a SuperPlot[83] with bold symbols representing the mean of an individual biological repeat, and the background symbols representing the data from individual cells. Statistical details, including n numbers and repeats can be found in the corresponding figure legend.

Current Biology, Volume *32*

Supplemental Information

A mechanism for exocyst-mediated tethering via Arf6 and PIP5K1C-driven phosphoinositide conversion

Hannes Maib and David H. Murray

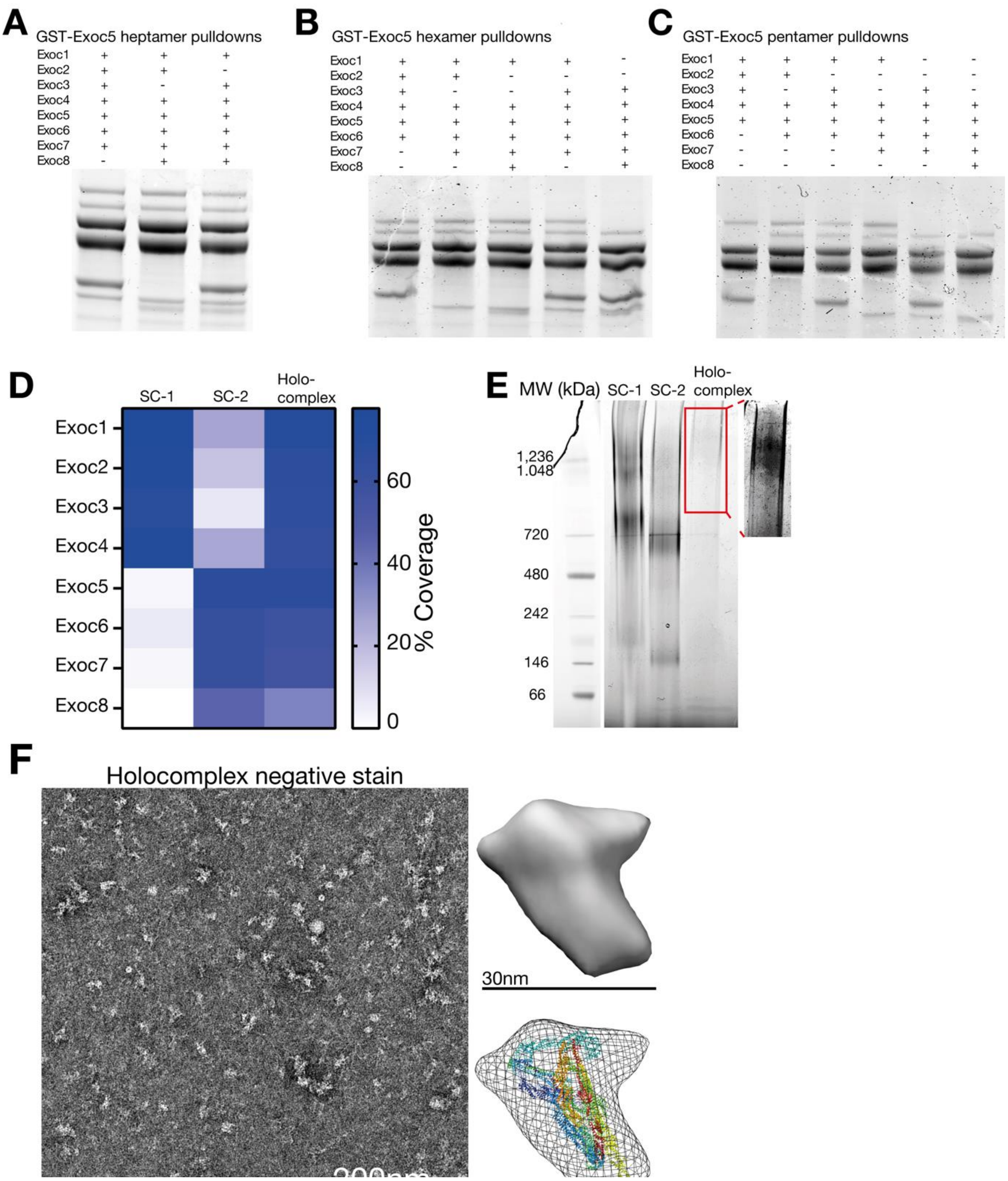

Figure S1. Exocyst connectivity and holocomplex verification and validation. Related to Figure 1
A-C, To confirm higher order exocyst complex connectivity, exocyst subunits were separately expressed in SF9 insect cells and mixed upon lysis. All possible combinations for exocyst hepta-, hexa-, and pentamers from pulldowns using Exoc5 tagged with GST and analysed by SDS-PAGE and Coomassie staining. **D**, Heatmap of protein coverage for mass spectrometry analysis of purified Subcomplex-1, -2 and holocomplex. **E**, Native gel electrophoresis of purified Subcomplex-1, -2 and holocomplex. Insert is shown in enhanced contrast to visualise faint bands from holocomplex. **F**, Exocyst holocomplex was analysed by negative stain and a low-resolution model was generated. The cryo-EM structure of the yeast exocyst (PDB ID 5YFP; ribbons) was placed into the outline (mesh) of this low-resolution shell for visual comparison.

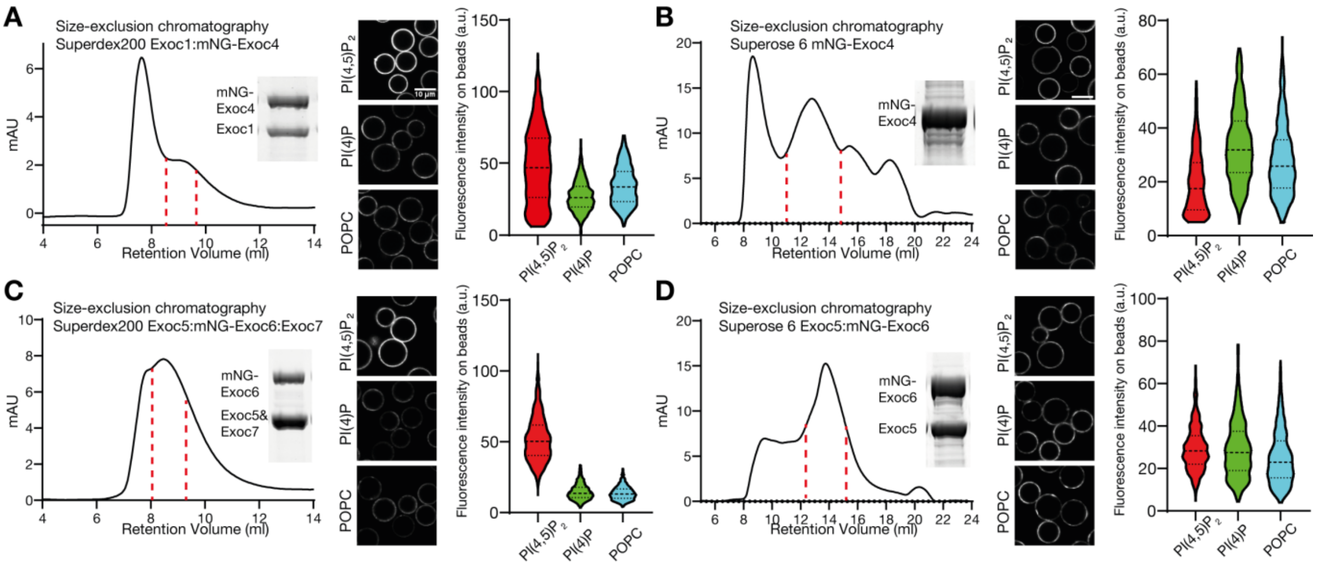

Figure S2. Purification and phosphoinositide binding of exocyst subcomplexes. **Related to Figure 2** **A**, Size-exclusion chromatography of a dimer of Exoc1 with mNeonGreen (mNG)-tagged Exoc4. **B**, Exoc1:4 dimer was added to membrane-coated beads. These were formed from a lipid composition of 84.9% 1-palmitoyl-2-oleoyl-glycero-3-phosphocholine (POPC), 10% phosphatidylserine and 5% phosphoinositide or POPC, doped with 0.1% rhodamine-DPPE. Individual beads were segmented, and mean fluorescent intensity was quantified in ImageJ. n=926-1589 beads. **C**, Size-exclusion chromatography of mNeonGreen (mNG) tagged Exoc4. **D,** Exoc4-mNG was added to membrane-coated beads as in B. n=745-881 beads. **E**, Size-exclusion chromatography of trimer of Exoc5:6:7 with mNeonGreen (mNG)-tagged Exoc6. **F**, Exoc5:6:7 timer was added to membrane-coated beads as in B. n=1032-1239 beads. **G**, Size-exclusion chromatography of dimer of Exoc5:6 with mNeonGreen (mNG) tagged Exoc6. **H**, Exoc5-6 dimer was added to membrane-coated beads as in B. n=1025-1178 beads.

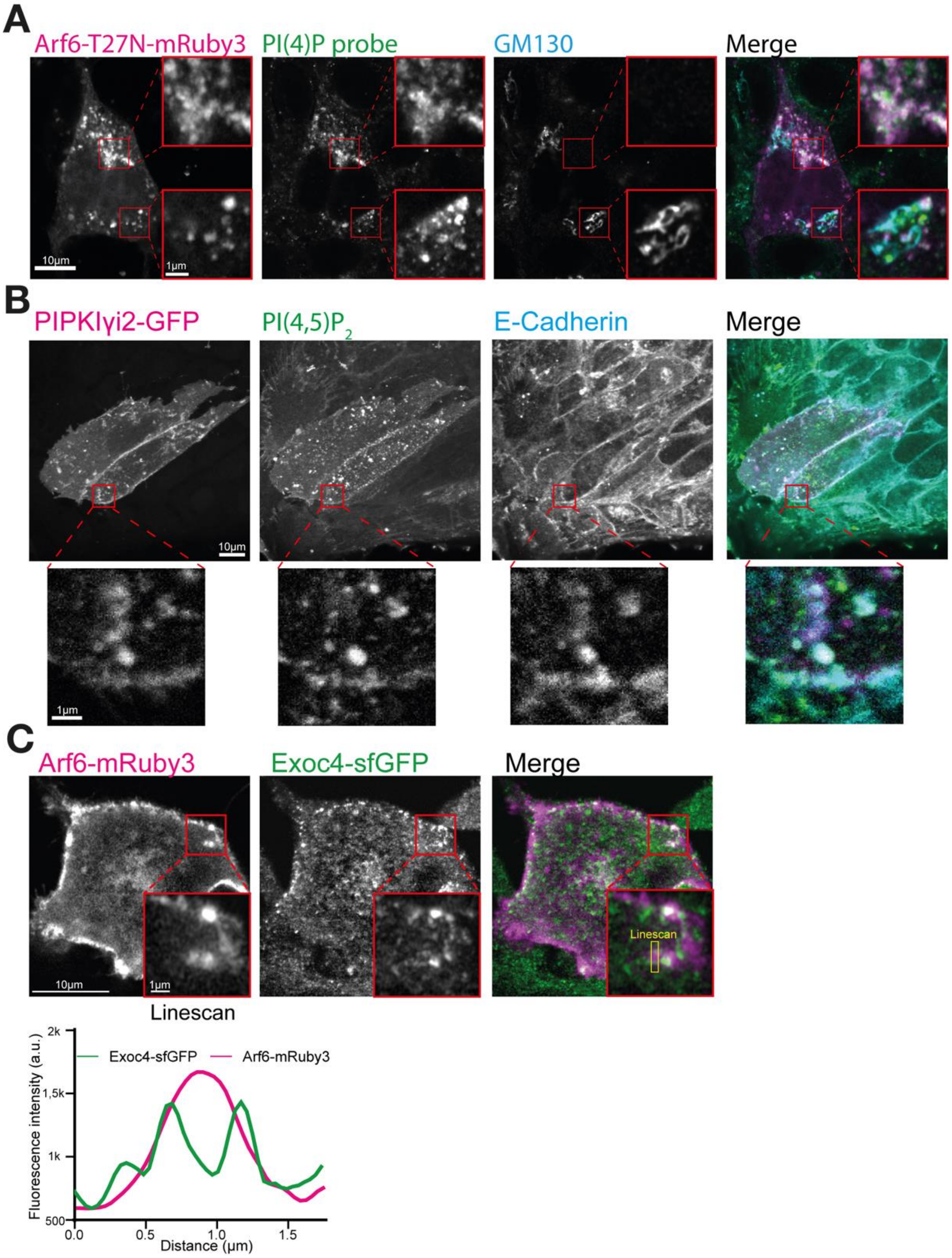

Figure S3. Subcellular localisation of phosphoinositides and Exoc4. Related to Figure 5
A, NMuMG cells were transfected with Arf6-T27N tagged with mRuby3 and stained for PI(4)P using recombinant SidC fused to GFP and conventional antibody staining against GM130. **B**, NMuMG cells were transfected with PIP5K1C tagged with EGFP and stained for PI(4,5)P_2 using recombinant PLCδ-PH fused to RFP, and conventional antibody staining against E-cadherin. **C,** NMuMG cells expressing Exoc4 with endogenous sfGFP tag were transfected with Arf6-WT tagged with mRuby3 and imaged by life cell Airyscan microscopy.
